# Is It Still Beneficial to Monitor the Trough Concentration of Vancomycin? A Quantitative Meta-Analysis of Nephrotoxicity and Efficacy

**DOI:** 10.3390/antibiotics13060497

**Published:** 2024-05-28

**Authors:** Wanqiu Yang, Kaiting Zhang, Yuancheng Chen, Yaxin Fan, Jing Zhang

**Affiliations:** 1 Institute of Antibiotics, Huashan Hospital, Fudan University, Shanghai 200040, China; 19111220010@fudan.edu.cn (W.Y.); 21111220006@m.fudan.edu.cn (K.Z.); 2 Key Laboratory of Clinical Pharmacology of Antibiotics, National Population and Family Planning Commission, Shanghai 200040, China; 3 National Clinical Research Center for Aging and Medicine, Huashan Hospital, Fudan University, Shanghai 200040, China; 4 Phase I Clinical Research Center, Huashan Hospital, Fudan University, Shanghai 200040, China; chenyuancheng@huashan.org.cn

**Keywords:** therapeutic drug monitoring, vancomycin, trough concentration, efficacy, nephrotoxicity

## Abstract

This study conducted a quantitative meta-analysis to investigate the association of vancomycin indicators, particularly area under the curve over 24 h (AUC_24_) and trough concentrations (C_trough_), and their relationship with both nephrotoxicity and efficacy. Literature research was performed in PubMed and Web of Science on vancomycin nephrotoxicity and efficacy in adult inpatients. Vancomycin C_trough_, AUC_24_, AUC_24_/minimum inhibitory concentration (MIC), nephrotoxicity evaluation and treatment outcomes were extracted. Logistic regression and *E*_max_ models were conducted, stratified by evaluation criterion for nephrotoxicity and primary outcomes for efficacy. Among 100 publications on nephrotoxicity, 29 focused on AUC_24_ and 97 on C_trough_, while of 74 publications on efficacy, 27 reported AUC_24_/MIC and 68 reported C_trough_. The logistic regression analysis indicated a significant association between nephrotoxicity and vancomycin C_trough_ (odds ratio = 2.193; 95% CI 1.582–3.442, *p* < 0.001). The receiver operating characteristic curve had an area of 0.90, with a cut-off point of 14.55 mg/L. Additionally, 92.3% of the groups with a mean AUC_24_ within 400–600 mg·h/L showed a mean C_trough_ of 10–20 mg/L. However, a subtle, non-statistically significant association was observed between the AUC_24_ and nephrotoxicity, as well as between AUC_24_/MIC and C_trough_ concerning treatment outcomes. Our findings suggest that monitoring vancomycin C_trough_ remains a beneficial and valuable approach to proactively identifying patients at risk of nephrotoxicity, particularly when C_trough_ exceeds 15 mg/L. C_trough_ can serve as a surrogate for AUC_24_ to some extent. However, no definitive cut-off values were identified for AUC_24_ concerning nephrotoxicity or for C_trough_ and AUC_24_/MIC regarding efficacy.

## 1. Introduction

Vancomycin is the first-line antibiotic for methicillin resistant *Staphylococcus aureus* (MRSA) infections [1], and is also used to treat suspected or confirmed infections caused by other Gram-positive bacteria. However, a narrow therapeutic index, which requires balancing efficacy with the risk of acute kidney injury (AKI), and large inter-patient variability in pharmacokinetics (PK) makes vancomycin dosing even more challenging, thus necessitating the use of therapeutic drug monitoring (TDM). 

Despite being in clinical use for over 60 years, there is still controversy regarding the most appropriate indicator and its respective target value to optimize vancomycin treatment and reduce toxicity. During the past few years, the ratio of area under the curve to minimum inhibitory concentration over 24 h (AUC_24_/MIC) has been advocated as the preferred parameter for measuring vancomycin’s effectiveness [1,2,3]. Due to difficulty in determining the AUC_24_ in routine clinical practice and subsequently calculating the AUC_24_/MIC, the 2009 American guideline suggested the trough concentration (C_trough_) as a surrogate marker for AUC_24_, which is recommended as the most accurate and practical method to monitor vancomycin [4]. However, with the development of new approaches, such as Bayesian software, estimating AUC_24_ has become more convenient. Some studies reported AUC_24_-guided dosing as more clinically effective [5,6,7] and having less risk of AKI over C_trough_-guide dosing [5,6,7,8,9,10,11]. Furthermore, C_trough_ was reported as not being substituted for AUC_24_ in some studies [12,13,14,15,16]. Thus, the 2020 American TDM guideline for vancomycin and the 2022 Japanese TDM guideline recommended AUC_24_/MIC as a reliable predictor to improve clinical efficacy and avoid nephrotoxicity, targeting a ratio of 400–600 [17,18].

However, some research still reported inconsistent results. Dalton et al. reported that a target AUC_24_/MIC index could not be established to achieve the optimal effectiveness and safety of vancomycin [19]. Bellos et al. discovered that an increase in C_trough_ was significantly associated with a higher risk of nephrotoxicity [20]. Lodise et al. found that vancomycin C_trough_ is the pharmacodynamic index that best describes the exposure–toxicity response relationship [21]. Moreover, recent studies provide growing evidence that C_trough_ is more strongly correlated with nephrotoxicity [22]. Meanwhile, resource-constrained settings that face challenges in estimating the AUC_24_ using a Bayesian approach or a first-order PK equation with two concentrations of steady-state samples remain prevalent, especially in developing countries [23]. Therefore, in some countries, not only is AUC_24_ still recommended, but C_trough_ is as well. For instance, the 2020 Chinese guideline suggests maintaining steady-state C_trough_ at 10–15 mg/L in adult patients and 10–20 mg/L in adult patients with serious MRSA infections [24]. The Anti-infectives Committee of the International Association of Therapeutic Drug Monitoring and Clinical Toxicology also recommends a target C_trough_ of 10–15 mg/L for serious MRSA infections in 2022 [25]. The European Society of Intensive Care Medicine recommended C_trough_ at 15–20 mg/L for severe infections in 2020 [26]. 

Although numerous reviews have examined a large number of clinical studies and proposed target values for the efficacy and nephrotoxicity of vancomycin, providing valuable clinical references, inconsistencies have persisted in previous meta-analysis. Meanwhile, traditional meta-analysis often faces significant heterogeneity among studies, encompassing differences in patient characteristics, definition of nephrotoxicity, and treatment outcomes. This can make it challenging to accurately assess the relationships and target values for vancomycin efficacy and nephrotoxicity.

Therefore, the present study employed a quantitative meta-analysis to investigate the relationship between vancomycin parameters (C_trough_ and AUC_24_ or AUC_24_/MIC) and both nephrotoxicity and efficacy, taking into account the varying definition of nephrotoxicity and treatment outcomes. This study aimed to evaluate the benefits of monitoring the C_trough_ and AUC_24_ of vancomycin, and to assess the relationship between AUC_24_ and C_trough_ in studies that included both measures.

## 2. Results

### 2.1. Characteristics of the Included Studies

**Figure 1:**
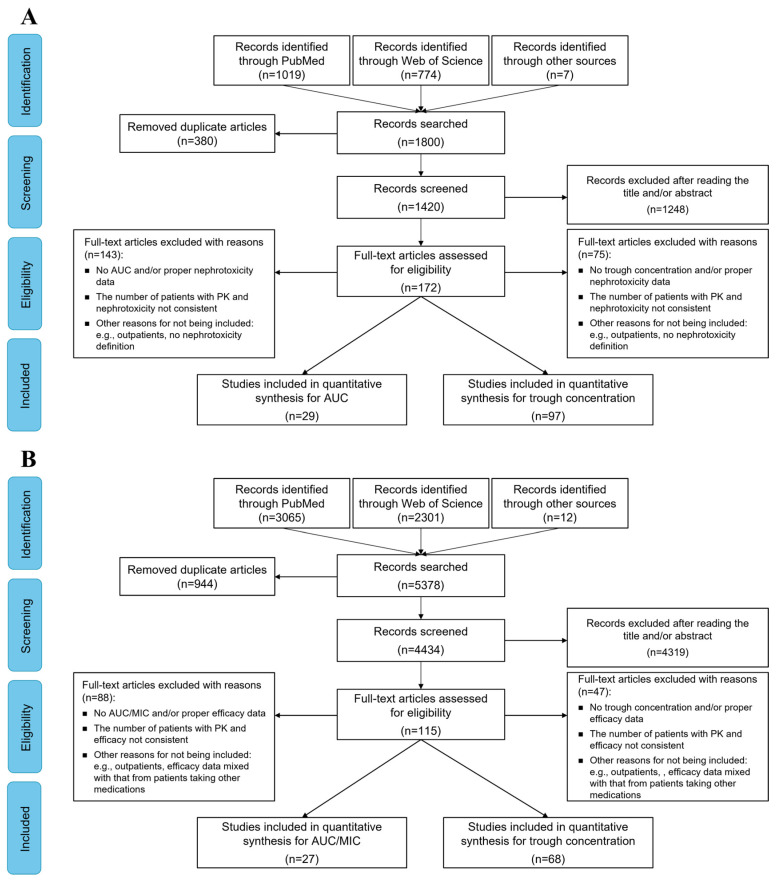
Flow chart for study identification and selection for nephrotoxicity (**A**) and efficacy (**B**).

A total of 172 from 1420 studies were subjected to further examination, and finally 100 articles (listed in the Supplementary Materials) were included in the nephrotoxicity analysis. Among them, 29 and 97 articles were included for target AUC_24_ and target C_trough_ evaluation, respectively (Figure 1A). Most of the studies adopted a retrospective design (n = 88), while 13 studies were prospective cohorts and 1 study conducted a post hoc analysis of randomized controlled trials (RCT). The 2009 consensus guideline [4] was the most commonly adopted criteria to define vancomycin nephrotoxicity (n = 65), while the Kidney Disease Improving Global Outcomes (KDIGO) [27], Acute Kidney Injury Network (AKIN) [28], and Risk, Injury, Failure, Loss of kidney function and End-stage kidney disease (RIFLE) [29] criteria were applied in 16, 14, and 16 studies, respectively.

A total of 115 from 4434 articles underwent detailed scrutiny for efficacy analysis. Finally, 74 articles (listed in the Supplementary Materials) were screened for inclusion in the efficacy analysis, of which 27 and 68 articles had the assessment of target AUC_24_/MIC and target C_trough_, respectively (Figure 1B). Out of the 74 studies, 61 adopted a retrospective design. The most frequently reported outcome was all-cause mortality (n = 55), followed by clinical failure (n = 27), microbiological failure (n = 26) and treatment failure (n = 25). The most commonly used methods for MIC testing were broth microdilution (BMD) (n = 16) and the Etest (n = 17) method, while agar dilution, VITEK 2 (https://www.biomerieux-usa.com/vitek-2, accessed on 24 April 2024) and MicroScan (https://www.beckmancoulter.com/products/microbiology/microscan-walkaway-plus-system, accessed on 24 April 2024) were used less frequently (n < 7).

Since 2012, the number of publications has significantly increased (ranging from 1990 to 2022), with the majority of results reported from United States, Japan and China. Further details on the study characteristics are provided in Supplementary Table S1 to Table S4.

### 2.2. Nephrotoxicity

**Figure 2:**
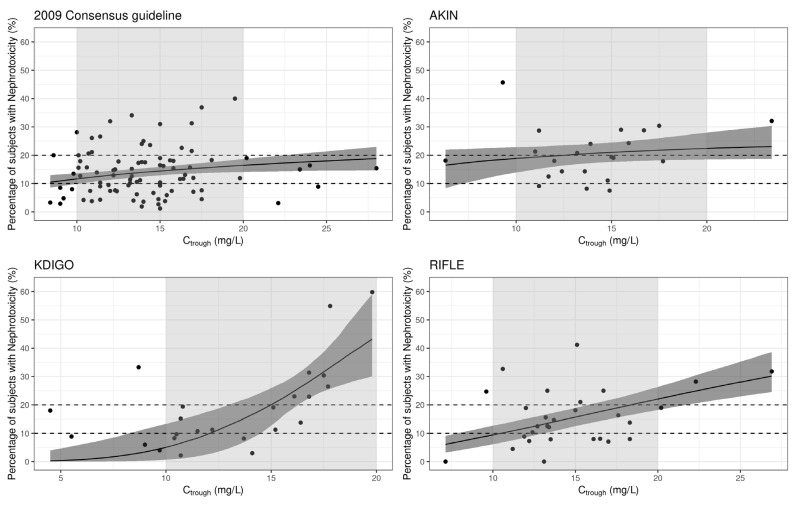
Correlation between nephrotoxicity and trough concentrations. Black circles represent the observed vancomycin C_trough_ values from each study. The solid line and the shaded area represent the estimated *E*_max_ model curve with 95% credible intervals of parameters. The grey shade represents the interval between C_trough_ 10 mg/L and 20 mg/L.

The non-linear association between vancomycin C_trough_ and the incidence of nephrotoxicity, stratified by different nephrotoxicity definition, is illustrated in Figure 2 and Table 1. The data indicate that a higher C_trough_ of vancomycin are associated with a higher incidence of nephrotoxicity, with a more obvious positive correlation observed for the KDIGO and RIFLE criteria. This relationship is also evident in the box plot of the probability of nephrotoxicity in different trough categories (Figure S1).

**Figure 3:**
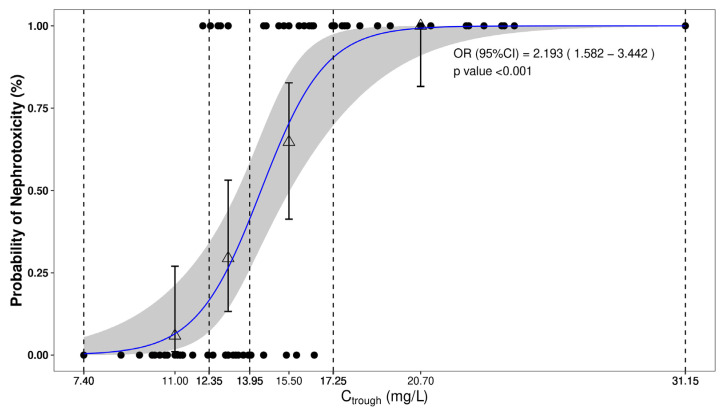
Logistic regression illustrating the association of the probability of experiencing nephrotoxicity and as a function of vancomycin trough concentrations. The upper and lower circles represent the presence or absence of a given nephrotoxicity across the range of vancomycin trough concentrations, respectively. The dots depict the observed incidence for the quartiles of exposure, whereas the corresponding vertical bars represent the exact 95% CI calculated using Wilson’s method. Finally, the middle line and its corresponding shaded area represent the model-based exposure–safety relationship and the 95% CI, respectively. Vertical dashed lines represent min, 25%, median, 75% and max percentile of trough concentrations, respectively.

The univariate logistic regression analysis (see Figure 3 for the 2009 consensus guidelines criteria) revealed that nephrotoxicity was significantly associated with vancomycin C_trough_ (OR (95%CI) = 2.193 (1.582–3.442), *p* < 0.001). The area under the receiver operating characteristic (ROC) curve (AUROC) value of 0.90 indicated the potential of vancomycin C_trough_ to serve as a predictor of vancomycin nephrotoxicity (Figure S2), with a cut-off of 14.55 mg/L, representing 79.4% sensitivity and 91.2% specificity in the study populations. Covariates with missing values less than 30% (i.e., age, serum creatinine and male percentage) were also evaluated for their association with nephrotoxicity, but none demonstrated a statistically significant relationship. A subgroup analysis of patients not receiving renal replacement therapy showed similar results (Figure S3).

**Figure 4:**
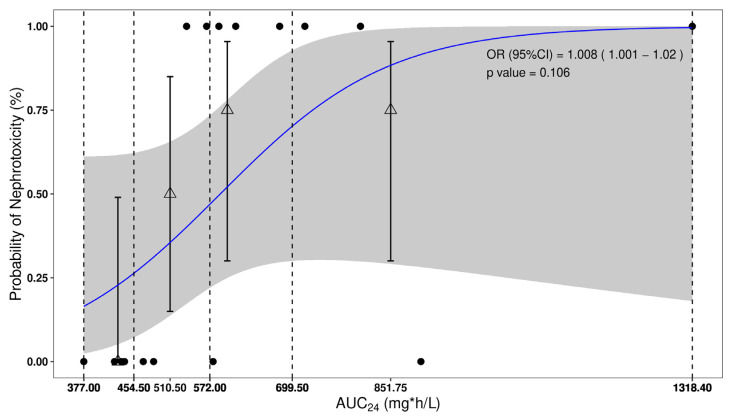
Logistic regression illustrating the association of the probability of experiencing nephrotoxicity and as a function of vancomycin AUC_24._ The upper and lower circles represent the presence or absence of a given nephrotoxicity across the range of vancomycin AUC_24_, respectively. The dots depict the observed incidence for the quartiles of exposure, whereas the corresponding vertical bars represent the exact 95% CI calculated using Wilson’s method. Finally, the middle line and its corresponding shaded area represent the model-based exposure–safety relationship and the 95% CI, respectively. Vertical dashed lines represent min, 25%, median, 75% and max percentile of AUC_24_, respectively.

A slight trend towards lower nephrotoxicity in patients with low AUC_24_ was observed in Figure S4. Logistic regression analysis examining the association between AUC_24_ and nephrotoxicity according to the 2009 consensus guideline, using data from eight articles, revealed a similar trend with a cut-off value of 510 mg·h/L, although the trend was not significant (OR = 1.008, 95%CI of 1.001–1.02, *p* > 0.05, Figure 4).

### 2.3. Efficacy

#### 2.3.1. Treatment Failure

**Figure 5:**
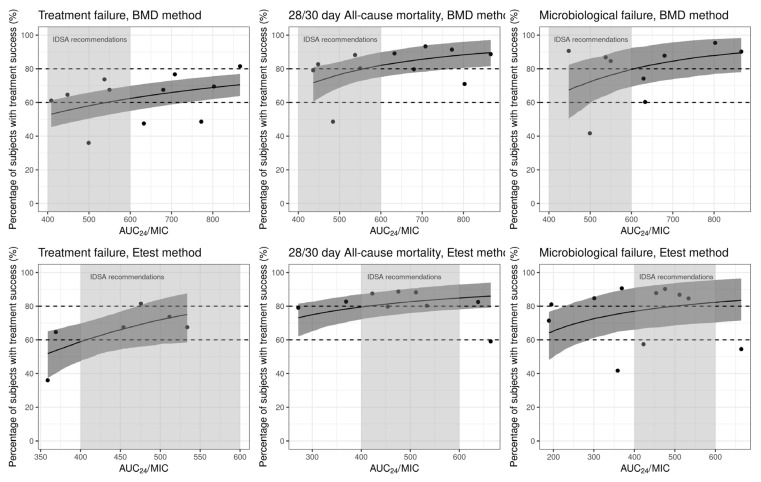
Correlation between outcomes and continuous AUC_24_/MIC stratified by MIC method. The solid black line and the shaded area represent the estimated *E*_max_ model curve with 95% credible intervals of parameters to reflect the correlation between clinical outcomes and AUC_24_/MIC. The black dashed transverse line represents the 60% and 80% treatment success rate for each clinical outcome. The black circles represent the observed incidence of success of each clinical outcome. The shaded area represents the AUC_24_/MIC interval between 400 and 600.

Eleven studies reported treatment failure as an outcome, with ten articles presenting results with BMD method and six using the Etest method. A subtle trend suggesting that a higher AUC_24_/MIC is associated with a lower treatment failure rate was observed (Figure 5, Table 1). When AUC_24_/MIC_BMD_ reached 400 or 600, the predicted treatment success rate was 52% and 62%, respectively. Similarly, when AUC_24_/MIC_Etest_ attained 400 or 600, the predicted treatment success rate was 59% and 80%. However, no statistically significant difference (OR = 1.017, 95% CI of 0.999–1.051, *p* > 0.05, Figure S5) in treatment success rates was identified across the range of AUC_24_/MIC_BMD_ in the logistic regression analysis. A similar trend was observed for the relationship between treatment failure rates and C_trough_ (Figure S6).

#### 2.3.2. All-Cause Mortality

Out of 18 studies that reported an all-cause mortality outcome, 12 studies reported the 30- or 28-day all-cause mortality, of which 8 articles reported results with BMD and the Etest method, respectively. Due to the limited sample size of studies reporting a binary outcome, only incidence was analyzed. A subtle trend emerged, suggesting a correlation between higher AUC_24_/MIC and lower 30-day all-cause mortality rates (Figure 5, Table 1). When the AUC_24_/MIC_BMD_ reached 400 or 600, the predicted survival rate was 76% and 83%, respectively. Similarly, when the AUC_24_/MIC_Etest_ reached 400 or 600, the predicted survival rate was 78% and 84%, respectively. A similar trend was observed for the relationship between 30-day all-cause mortality rate and C_trough_ (Figure S6).

#### 2.3.3. Microbiologic Failure

Of 17 studies reporting microbiologic failure, 9 articles utilized BMD method and the Etest method, respectively. Microbiologic failure appeared to be lower in patients with higher AUC_24_/MIC and C_trough_ (Figure 5, Table 1 and Figure S6). The AUC_24_/MIC_BMD_ and AUC_24_/MIC_Etest_ equal to 400 resulted in a microbiologic success rate of 63% and 77%, respectively.

Due to the limited number of articles, it is not possible to analyze clinical failure outcomes. In the subgroup of patients with MRSA infections, the trends observed in the above analysis were similar (results not shown). This suggests that the relationship between AUC_24_/MIC and clinical outcomes may be consistent in this patient population.

### 2.4. Relationship of Vancomycin Mean AUC_24_ and C_trough_

**Figure 6:**
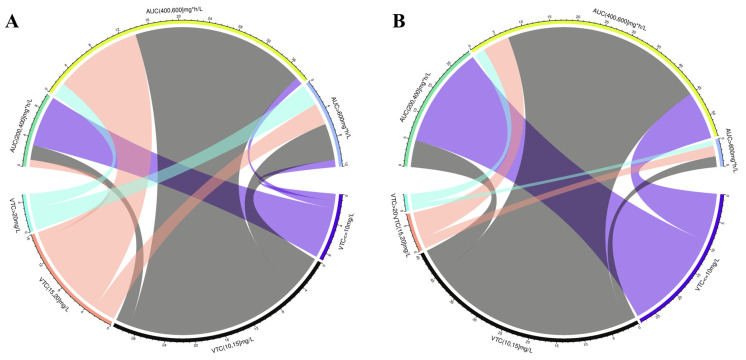
Correlation between exposure metrics (Steady-state AUC_24_ and trough concentrations). The chord diagram presents the difference of the mean VTC with mean AUC_24_ for each subgroup of studies. (**A**) The correlation between AUC_24_ and trough concentration from studies included in the nephrotoxicity analysis; (**B**) the correlation between AUC_24_ and trough concentration from studies reporting efficacy. VTC: vancomycin trough concentration.

The mean C_trough_ values were categorized into the following groups: ≤10 mg/L, 10–15 mg/L, 15–20 mg/L and >20 mg/L. Similarly, the mean AUC_24_ were divided into ≤200 mg·h/L, 200–400 mg·h/L, 400–600 mg·h/L and >600 mg·h/L. The chord diagram vividly demonstrates the relationship between the mean C_trough_ and mean AUC_24_ for each subgroup of studies (Figure 6, Table 2).

Among the paired groups (n = 61) from studies included in the nephrotoxicity analysis, 77.8% of the groups with mean C_trough_ ≤ 10 mg/L had mean AUC_24_ < 400 mg·h/L, while all groups with mean C_trough_ > 20 mg/L (n = 5) had AUC_24_ > 400 mg·h/L, among which 60% had AUC_24_ values > 600 mg·h/L. In the subgroups with mean AUC_24_ within 400–600 mg·h/L, 92.3% had mean C_trough_ of 10–20 mg/L.

Of the paired groups (n = 83) from studies included in the efficacy analysis with both exposure measures, 67.9% of the groups with mean C_trough_ ≤ 10 mg/L had mean AUC_24_ < 400 mg·h/L, while all groups with C_trough_ > 20 mg/L (n = 3) had AUC_24_ > 400 mg·h/L. When C_trough_ reached 10–15 mg/L, the rate of vancomycin AUC_24_ in 400–600 mg·h/L was 84.4%, and when C_trough_ reached 15–20 mg/L, the rate of vancomycin AUC_24_ in 400–600 mg·h/L was 71.4%. In other words, 79.7% of the subgroups with mean AUC_24_ within 400–600 mg·h/L had mean C_trough_ of 10–20 mg/L.

## 3. Discussion

The recent American and Japanese TDM guidelines recommend AUC_24_/MIC as the preferred approach for enhancing vancomycin efficacy and educing nephrotoxicity, and C_trough_ is no longer recommended [17,18]. Nevertheless, obtaining timely and accurate AUC_24_ poses challenges, and measuring C_trough_ remains the most efficient and accessible method to monitor vancomycin dosing, especially in source-limited settings. Therefore, the question of whether C_trough_ monitoring remains beneficial warrants further investigation.

Our quantitative meta-analysis demonstrated a robust correlation between vancomycin C_trough_ and nephrotoxicity (OR (95%CI) = 2.193 (1.582–3.442), *p* < 0.001), with a cut-off point identified at 14.55 mg/L. This finding aligns with the recommendations of a clinical guideline [24] and a position statement [25]. Moreover, our analysis revealed that studies with a mean C_trough_ of 10–15 mg/L and 15–20 mg/L showed that almost 80% had a mean AUC_24_ 400–600 mg·h/L, suggesting that C_trough_ can serve as a surrogate for AUC_24_ to some degree. This is further supported by a recent multicenter, retrospective study in China that focused on critically ill patients without any form of dialysis [23]. Our study supports the clinical utility of C_trough_ monitoring, particularly for nephrotoxicity prevention, as it correlates with AUC_24_ within specific ranges. However, the direct substitution of C_trough_ for AUC_24_ is not always feasible due to individual pharmacokinetic variations. Developing an AUC_24_-C_trough_ equation could establish patient-specific C_trough_ targets for individualized management.

In addition, our study revealed that the application of KDIGO and RIFLE criteria for assessing kidney toxicity yielded higher sensitivity in identifying vancomycin-associated nephrotoxicity compared to the 2009 consensus guideline. This disparity in sensitivity could be attributed to the slightly higher threshold defined in the 2009 guideline (an increase in the serum creatinine ≥0.5 mg/dL [4]), which has been updated in the latest guideline [17]. 

Unfortunately, although we observed a subtle trend that higher C_trough_ were associated with lower treatment or microbiologic failure rates and 30-day all-cause mortality rates, no definitive cut-off value was identified. This lack of specificity can be attributed to the fact that the majority of C_trough_ falls within the common therapeutic range of 10–20 mg/L for vancomycin. However, considering the observed efficacy outcome (Figure S6) and the simulation using the *E*_max_ model, it appears that a C_trough_ greater than 15 mg/L nearly reaches the plateau of the efficacy curve.

Furthermore, we evaluated the relationship between AUC_24_ and vancomycin nephrotoxicity and the relation between AUC_24_/MIC and efficacy outcome. While a subtle trend of reduced nephrotoxicity in patients with lower AUC_24_ was observed, it did not reach statistical significance. This could be attributed to the fact that only eight included studies with a mean AUC_24_ were centered on the range of 400–600 mg·h/L. However, we attempted to identify a cut-off value at 510 mg·h/L. Concerning the correlation between AUC_24_/MIC and the efficacy outcome, our analysis revealed that when the AUC_24_/MIC_Etest_ and AUC_24_/MIC_BMD_ exceeded 500–600, both the treatment/microbiologic success rate and 30-day survival rates appeared to approach the efficacy curve plateau. Nonetheless, these findings should be interpreted with caution given the narrow range of AUC_24_/MIC values obtained from clinical settings employing TDM. In summary, AUC_24_ not exceeding 500 mg·h/L (assuming the MIC as 1 mg/L) may favor both clinical efficacy and nephrotoxicity avoidance.

There are some limitations for the selected studies. The most common limitation the selected studies mentioned is the retrospective nature of the study design, which is also one of limitations of our analysis, i.e., the majority of the included studies (greater than 80%) were retrospective, which introduces a risk of unmeasured confounding effects and bias. In addition, in the selected studies, the small sample size of the study, a single center being included in most studies, and the fact that a limited type of patient population hinders extrapolation to a wider range of people were also mentioned. However, what we conducted was a quantitative meta-analysis including all the patient population data for analysis, which addressed the concern of the small sample size, single center and single type patient population in each study. Furthermore, some other limitations for our analysis needed to be considered when interpreting the results. Firstly, articles reported different detection methods for vancomycin concentration (most are commercial immunoassays), along with various types of C_trough_ measurements, including initial or first steady-state values, average, highest or predicted values used in each article, which may introduce bias. Additionally, among different studies, the severity of the disease and the physiological and pathological condition of the patients vary, and the limited data availability hindered the evaluation of covariates on nephrotoxicity or efficacy, such as co-administered medication, renal function (creatinine clearance rate and renal replacement therapy), and critically ill patients’ percentage. Finally, most studies published after 2009 focused on collecting data within the recommended range of vancomycin C_trough_ and AUC_24_ due to the widespread use of TDM. The narrow range of data might obscure the relationship for the two indictors, making it challenging to draw definitive conclusions.

## 4. Methods

### 4.1. Search Strategy

The literature search was performed using the PubMed and Web of Science database. The search keywords for the analysis of association between exposure and nephrotoxicity included “vancomycin”, its exposure parameters (“area under the concentration-time curve”, “trough concentration”, “exposure”, “pharmacokinetics” and “pharmacokinetics /pharmacodynamics”) and safety related indicators (“nephrotoxicity”, “acute kidney injury”, “renal failure”, “renal impairment”). 

Similarly, for the analysis of association between exposure and efficacy, the search keywords for vancomycin-related ones included the above mentioned keywords and also “area under the concentration-time curve to minimum inhibitory concentration ratio”, while efficacy related indicators included “efficacy”, “clinical outcome”, “clinical failure”, “clinical response”, “microbiological failure”, “treatment failure”, “success”, “mortality” and “eradication”. 

The reference lists of the included studies and historical systematic reviews were searched using a snowball method to identify potential additional sources. No language or date restrictions were imposed, but the patients were limited to adults.

### 4.2. Inclusion Criteria and Outcomes

We included adult inpatients treated with intravenous vancomycin and studies from RCT, as well as prospective and retrospective studies that met the searching criteria.

The inclusion criteria for the analysis of the association between vancomycin exposure (AUC_24_ and C_trough_) and nephrotoxicity included studies reporting AUC_24_ or/and C_trough_, along with detailed definitions of nephrotoxicity events. The primary outcome was the incidence of nephrotoxicity. Likewise, for the association between a vancomycin indicator (AUC_24_/MIC or C_trough_) and efficacy, the inclusion criteria included studies reporting AUC_24_/MIC or/and C_trough_ and respective outcomes, i.e., treatment failure, all-cause mortality, microbiologic failure, or clinical failure. The primary outcome was treatment failure and 30- or 28-day all-cause mortality. Secondary outcomes were microbiologic failure and clinical failure. Treatment failure was defined as any combination of death, clinical non-improvement or worsening, need for antibiotic modification, microbiologic failure or recurrence of bacteremia. No specific patient populations or infections were excluded.

### 4.3. Data Extraction

The analysis of nephrotoxicity in the extraction of data comprised the following information: characteristics of the literature (year of publication, name of first author, region or country of study); study design (trial type, eligibility criteria, patient population, and sample size); study outcomes of vancomycin exposure (measurement of AUC_24_ or C_trough_, method of AUC_24_ calculation, timing of AUC_24_ calculation and C_trough_ collection relative to start of therapy) and nephrotoxicity (continuous (incidence rate) and/or binary (yes vs. no) nephrotoxicity outcome) per different evaluation criterion like the 2009 vancomycin consensus [4], KDIGO [27], AKIN [28] or RIFLE [29] guidelines), and patient characteristics (age, weight, renal function, proportion of male patients, coadministration of nephrotoxins and critically ill /intensive care unit status).

For the efficacy analysis, the data extraction involved literature characteristics, the study design as mentioned above, study outcomes of exposure parameters (measurement of AUC_24_/MIC or C_trough_, method of AUC_24_ calculation, timing of AUC_24_ calculation and C_trough_ collection relative to start of therapy, method of MIC determination) and efficacy (the continuous and/or binary clinical outcome measures), and patient characteristics (age, weight, renal function, proportion of male patients and critically ill/intensive care unit status).

Data extraction was conducted independently by two authors who applied the inclusion criteria. In case of any disagreements, alignment was achieved through consensus.

### 4.4. Data Handling

Two types of outcomes (all-cause mortality, treatment failure, microbiologic failure, clinical failure and nephrotoxicity) were collected, i.e., proportion (incidence rate) and binary variables (yes or no). The mean values of AUC_24_/MIC, AUC_24_ and C_trough_ were extracted and treated as continuous variables, except for the articles that only reported median values, which were used instead. 

The nephrotoxicity outcome was analyzed, stratified by the evaluation criterion (i.e., 2009 vancomycin consensus, KDIGO, AKIN and RIFLE guidelines). A subgroup analysis of patients in the intensive care unit and without receipt of dialysis was also performed separately, provided that enough studies (>5) were available.

The efficacy outcomes, including all-cause mortality, treatment failure, microbiologic failure and clinical failure, were analyzed separately. To account for potential variations in MIC results due to the use of different MIC testing methods, the analysis was stratified by the MIC testing method. Due to the small sample size of articles reporting MIC testing methods of agar dilution, VITEK 2 and MicroScan, only articles reporting BMD and the Etest method were included. Additionally, a subgroup analysis of only MRSA-infected individuals was performed separately.

### 4.5. Analytical Method

An exploratory analysis revealed a trend of gradually increasing outcome proportions along with increasing vancomycin indicators, reaching a plateau at higher levels. The distributional characteristics of these data were described by the *E*_max_ model (Equation (1)). A fit-for-purpose simulation using the typical values of parameters from the *E*_max_ model was conducted to obtain the incidence of outcome at a certain value of C_trough_ or AUC_24_:

(1) $$E=\frac{E_{max}\cdot C^{\gamma }}{{EC}_{50}^{\gamma }+C^{\gamma }}$$

where *E*_max_ represents the maximum effect, while *EC*_50_ represents the indicators required to achieve half the *E*_max_. The slope factor (also known as Hill factor), represented by γ, measures the sensitivity of the response to the indicator’s change, determining the steepness of the curve.

Binary outcomes were analyzed using a logistic regression model (Equation (2)). A univariate logistic regression analysis was first performed to assess the relationship between the vancomycin indicators (as a continuous variable) and the outcomes. Optimal cut-off points were derived from the ROC curves using Youden’s index [30]. The study employed a univariate logistic regression analysis to evaluate patient characteristics, such as age and renal function, as potential factors influencing the outcome. Missing data were imputed by using the median value of the entire study population, and variables with missing proportions exceeding 30% were excluded from evaluation. A corresponding odds ratio (OR) in relation to the reference group, along with the 95% confidence interval (CI) and *p*-values, were calculated for each univariate logistic regression model. Variables with a *p* value of <0.05 in the univariate analysis were included in the multivariate analysis. To evaluate the discrimination of the logistic regression model, ROC curves were constructed, and AUROC was calculated as follows:

(2) $$\log\left[\frac{p}{1-p}\right]=\beta_{0}+\beta_{1}X_{1}+\cdots +\beta_{k}X_{k}$$

where p is the probability that an observation is in a specified category of the binary Y variable, $\frac{p}{1-p}$ describes the odds of being in the current category of interest, the (natural) logarithm of the odds $\log\left[\frac{p}{1-p}\right]$ is a linear function of the X variables (and is often called the log odds). This is also referred to as the logit transformation of the probability of success. *β*_0_ is the coefficient on the constant term, X is the independent variable(s), and $\beta_{k}$ is the coefficient on the $k^{th}$ independent variable.

The data management, all the analysis, simulation and plotting were carried out using the R software (version 4.2.0, Comprehensive R Network, http://cran.r-project.org/, accessed on 9 December 2023).

## 5. Conclusions

In conclusion, our quantitative meta-analysis has provided evidence of the correlation between vancomycin C_trough_ and nephrotoxicity incidence. The findings support that monitoring C_trough_ is still beneficial and can be a valuable approach in clinical practice, particularly when the concentration exceeds 15 mg/L. C_trough_ can serve as a surrogate for AUC_24_ to some extent. No definite cut-off was determined for AUC_24_ in relation to nephrotoxicity, and likewise, for C_trough_ and AUC_24_/MIC in terms of efficacy, underscoring the need for additional investigations.

## Figures and Tables

**Table 1:** Estimated parameters of *E*_max_ model for nephrotoxicity and efficacy endpoints.

Outcome	Endpoint	PK/PD Parameter	*E*_max_ (%) (CV%)	*EC*_50_ (mg/L) (CV%)	γ (CV%)
Nephrotoxicity	2009 Consensus	C_trough_ (mg/L) (n = 90)	32.5 (43.7%)	18.8 (78.4)	1.0 (FIX)
	AKIN	C_trough_ (mg/L) (n = 22)	42.7 (37.6)	21.4 (86.2)	1.51 (148)
	KDIGO	C_trough_ (mg/L) (n = 24)	100 (FIX)	22.7 (19.8)	4.15 (45.1)
	RIFLE	C_trough_ (mg/L) (n = 29)	100 (FIX)	51.1 (25.1)	1.47 (23.1)
Efficacy	Treatment failure	AUC_24_/MIC_BMD_ (n = 11)	100 (FIX)	367 (20.0)	1.0 FIX
		AUC_24_/MIC_Etest_ (n = 6)	100 (FIX)	335 (36.6)	2.65 (59.2)
	30- or 28-day all-cause mortality	AUC_24_/MIC_BMD_ (n = 8)	100 (FIX)	123 (19.4)	1.0 FIX
		AUC_24_/MIC_Etest_ (n = 9)	100 (FIX)	96.7 (62.3)	1.03 (54.4)
	Microbiologic failure	AUC_24_/MIC_BMD_ (n = 9)	100 (FIX)	296 (36.0)	2.39 (64.9)
		AUC_24_/MIC_Etest_ (n = 11)	100 (FIX)	99.8 (60.8)	1.01 (79.2)

AKIN = Acute Kidney Injury Network; KDIGO = Kidney Disease Improving Global Outcomes; RIFLE = Risk, Injury, Failure, Loss of kidney function and End-stage kidney disease; BMD = broth microdilution; CV = coefficient of variation; PK/PD = pharmacokinetic/pharmacodynamics; *E*_max_ = maximum effect; *EC*_50_ = the indicators required to achieve half the *E*_max_; γ = slope factor (also known as Hill factor).

**Table 2:** The overall distribution of mean trough concentration and AUC_24_.

Analysis, n (%/%)		C_trough_ (mg/L)			
Nephrotoxicity (n = 61)	AUC_24_ (mg·h/L)	≤10 (n = 9)	10–15 (n = 31)	15–20 (n = 16)	>20 (n = 5)
	≤200 (n = 0)	0 (0/0)	0 (0/0)	0 (0/0)	0 (0/0)
	200–400 (n = 10)	7 (70.0/77.8)	2 (20.0/6.5)	1 (10.0/6.2)	0 (0/0)
	400–600 (n = 39)	1 (2.6/11.1)	24 (61.5/77.4)	12 (30.8/75.0)	2 (5.1/40.0)
	>600 (n = 12)	1 (8.3/11.1)	5 (41.7/16.1)	3 (25.0/18.8)	3 (25.0/60.0)
Efficacy (n = 83)	AUC_24_ (mg·h/L)	≤10 (n = 28)	10–15 (n = 45)	15–20 (n = 7)	>20 (n = 3)
	≤200 (n = 0)	0 (0/0)	0 (0/0)	0 (0/0)	0 (0/0)
	200–400 (n = 24)	19 (79.2/67.9)	5 (20.8/11.1)	0 (0/0)	0 (0/0)
	400–600 (n = 54)	9 (16.7/32.1)	38 (70.4/84.4)	5 (9.3/71.4)	2 (3.7/66.7)
	>600 (n = 5)	0 (0/0)	2 (40.0/4.4)	2 (40.0/28.6)	1 (20.0/33.3)

n (%/%) represents the numbers of groups in each paired group with the percentage (the first %) of groups in each of the AUC_24_ categories and the percentage (the second %) of groups in each of the C_trough_ categories.

## Data Availability

The data presented in this study are available on request from the corresponding author.

## Supplementary Materials

The following supporting information can be downloaded at: https://www.mdpi.com/article/10.3390/antibiotics13060497/s1, Table S1. Methodological characteristics of the included studies for nephrotoxicity; Table S2. Patients’ characteristics in included studies for nephrotoxicity; Table S3. Methodological characteristics of the included studies for efficacy; Table S4. Patients’ characteristics in included studies for efficacy. Figure S1. Probability of nephrotoxicity for the trough category; Figure S2. Receiver operating characteristic curve of predictive level of vancomycin trough concentration for nephrotoxicity; Figure S3. Logistic regression illustrating the association of the probability of experiencing nephrotoxicity and as a function of vancomycin trough concentrations in patients without any form of dialysis; Figure S4. Correlation between nephrotoxicity and AUC_24_; Figure S5. Logistic regression illustrating the association of the treatment success rates and as a function of vancomycin AUC_24_/MIC_BMD_; Figure S6. Correlation between efficacy outcomes and trough levels. References [31,32,33,34,35,36,37,38,39,40,41,42,43,44,45,46,47,48,49,50,51,52,53,54,55,56,57,58,59,60,61,62,63,64,65,66,67,68,69,70,71,72,73,74,75,76,77,78,79,80,81,82,83,84,85,86,87,88,89,90,91,92,93,94,95,96,97,98,99,100,101,102,103,104,105,106,107,108,109,110,111,112,113,114,115,116,117,118,119,120,121,122,123,124,125,126,127,128,129,130,131,132,133,134,135,136,137,138,139,140,141,142,143,144,145,146,147,148,149,150,151,152,153,154,155,156,157,158,159,160,161,162,163] are cited in Supplementary Materials.
