# Supplementary material for: Is It Still Beneficial to Monitor the Trough Concentration of Vancomycin? A Quantitative Meta-Analysis of Nephrotoxicity and Efficacy
Source: Antibiotics (Basel). 2024 May 28;13(6):497. doi: 10.3390/antibiotics13060497 (PMC11200798; doi:10.3390/antibiotics13060497)
Supplement: Supplementary file 1 [file antibiotics-13-00497-s001.zip › antibiotics-3003639-supplementary.pdf]

1: SUPPLEMENTARY TABLES

Table S1. Methodological characteristics of the included studies for Nephrotoxicity

Year; Author	Sample size	Study design	Country/ Region	Setting	AKI definition	Trough Level (Y/NS)	AUC_{24h} (Y/NS)	Key Exclusion criteria
2021; Sohn [1]	37	Retrospective	Korea	Hospital/ICU	2009 Consensus	NS	Y	polymicrobial bacteremia
2021; Marko [2]	128	Retrospective	Canada	Hospital/ICU	2009 Consensus	Y	NS	RRT
2021; Katip [3]	312	Retrospective	Thailand	Hospital	2009 Consensus	Y	NS	polymicrobial infection, RRT
2019; Al-Sulaiti [4]	65	RCT	Qatar	Hospital/ICU	2009 Consensus	Y	Y	renal instability (history of AKI), end-stage renal disease
2018; Wan [5]	103	Retrospective	Canada	Hospital/ICU	RIFLE	Y	NS	RRT
2018; Mogle [6]	46	Retrospective	USA	Hospital/ICU	2009 Consensus	NS	Y	history of AKI, RRT, baseline serum creatinine >2 mg/dL, polymicrobial bacteremia, other anti-MRSA treatment
2018; Jumah [7]	57	Retrospective	Singapore	Hospital/ICU	2009 Consensus	Y	Y	RRT, polymicrobial bacteremia
2016; Fukumori [8]	81	Retrospective	Japan	Hospital	2009 Consensus	Y	Y	RRT, CLcr < 10 mL/min, weight < 25 kg
2015; Suzuki [9]	63	Retrospective	Japan	Hospital	2009 Consensus	Y	NS	RRT, other anti-MRSA treatment, Gram-negative bacteremia
2020; Zhang [10]	57	Retrospective	China	ICU	KDIGO	Y	NS	CLcr <50 mL/min
2020; Perin [11]	179	Retrospective	France	ICU	KDIGO	Y	NS	RRT
2019; Yahav [12]	285	Retrospective	Israel	Hospital/ICU	RIFLE	Y	NS	Polymicrobial infections
2017; Frazee [13]	399	Prospective	USA	ICU	KDIGO	Y	NS	RRT, history of AKI, eCLcr<20 mL/min, weight<40 kg, body mass index<40 kg/m^2
2012; Suzuki [14]	31	Retrospective	Japan	Hospital	2009 Consensus	Y	Y	RRT, other anti-MRSA treatment
2001; Wysocki [15]	119	Prospective	France	ICU	2009 Consensus	Y	Y	b-lactam allergy
2013; Gawronski [16]	59	Retrospective	USA	Hospital/ICU	2009 Consensus	Y	NS	RRT, polymicrobial bacteremia
2016; Duszynska [17]	42	Prospective	Poland	ICU	AKIN	NS	Y	RRT, history of AKI
2013; Mizokami [18]	94	Retrospective	Japan	Hospital	2009 Consensus	Y	Y	-
2017; Zasowski [19]	323	Retrospective	USA	Hospital	2009 Consensus	Y	Y	RRT, baseline serum creatinine >=2 mg/dL, history of AKI, concomitant piperacillin-tazobactam
2017; Finch [20]	1280	Retrospective	USA	Hospital	2009 Consensus/AKIN/RIFLE	Y	Y	RRT, baseline serum creatinine >=2 mg/dL, concomitant piperacillin-tazobactam
2022; Wang [21]	64	Retrospective	China	ICU	2009 Consensus	Y	Y	RRT, baseline serum creatinine >=2 mg/dL

Year; Author	Sample size	Study design	Country/ Region	Setting	AKI definition	Trough Level (Y/NS)	AUC_{24h} (Y/NS)	Key Exclusion criteria
2022; Ueda [22]	260	Retrospective	Japan	Hospital/ICU	2009 Consensus	Y	Y	RRT, other anti-MRSA treatment, concomitant nephrotoxic antimicrobial agents
2021; Yasu [23]	122	Retrospective	Japan	Hospital	KDIGO	Y	NS	-
2021; Wang [24]	349	Retrospective	China	Hospital	2009 Consensus	Y	NS	RRT, CLcr<20 mL/min, history of AKI
2021; Liu [25]	752	Retrospective	China	Hospital/ICU	KDIGO	Y	NS	RRT, eGFR<30 mL/min/1.73m 2, serum creatinine > 1.5-fold baseline
2021; Johnston [26]	89	Retrospective	USA	Hospital/ICU	2009 Consensus	Y	Y	RRT, other anti-MRSA treatment
2021; Al Sulaiman [27]	210	Retrospective	Saudi Arabia	ICU	AKIN	Y	NS	RRT, non-ICU
2020; Zhang [28]	168	Prospective	China	Hospital	2009 Consensus/RIFLE	Y	NS	RRT, eGFR <30 mL/min/1.73 m^2
2020; Ueki [29]	148	Retrospective	Japan	Hospital/ICU	2009 Consensus	Y	NS	RRT
2020; Qin [30]	9305	Retrospective	China	Hospital	KDIGO	Y	NS	treatment < 3 days
2020; Pan [31]	204	Retrospective	China	Hospital/ICU	KDIGO	Y	NS	RRT
2020; Mcgrady [32]	264	Retrospective	USA	Hospital	RIFLE	Y	Y	ICU, RRT, unstable renal function
2020; Ma [33]	127	Retrospective	Canada	Hospital	RIFLE	NS	Y	RRT
2020; Imai [34]	1141	Retrospective	Japan	Hospital/ICU	2009 Consensus	Y	NS	RRT, history of AKI
2020; Brunetti [35]	408	Retrospective	Korea	Hospital	2009 Consensus	Y	Y	RRT, CLcr<40 mL/min
2018; Truong [36]	100	Retrospective	USA	ICU	RIFLE	Y	Y	RRT
2019; Yahav [37]	285	Retrospective	Israel	Hospital	RIFLE	Y	NS	ICU
2019; de Almeida [38]	98	Prospective	Brazil	Hospital	KDIGO	Y	NS	ICU, RRT, eGFR <30 mL/min/1.73m^2
2018; Nakashima [39]	152	Retrospective	Japan	Hospital	2009 Consensus	Y	NS	RRT
2018; May [40]	165	Retrospective	USA	ICU	AKIN	Y	NS	RRT, SCr >2.0 mg/dL, neutropenia
2018; Liang [41]	370	Prospective	China	Hospital/ICU	KDIGO	Y	NS	gram-positive bacteria colonization, linezolid or teicoplanin treatment
2017; Han [42]	270	Retrospective	USA	Hospital/ICU	2009 Consensus	Y	NS	RRT
2017; Chavada [43]	127	Retrospective	Australia	Hospital/ICU	2009 Consensus	Y	Y	RRT
2017; Anderson [44]	455	Retrospective	USA	Hospital	AKIN	Y	NS	ICU, RRT, SCr >=1.5 mg/dL
2016; Hammoud [45]	498	Retrospective	USA	Hospital	2009 Consensus	Y	NS	RRT, history of AKI
2015; Hanrahan [46]	158	Retrospective	Australia	ICU	RIFLE	Y	NS	RRT, history of AKI
2015; Dong [47]	90	Retrospective	China	ICU	2009 Consensus/RIFLE	Y	NS	RRT, CLcr<30 mL/min, receiving concomitant nephrotoxins
2014; Hanrahan [48]	1430	Retrospective	UK	ICU	RIFLE	Y	NS	-
2014; Hall [49]	92	Retrospective	USA	Hospital/ICU	2009 Consensus	Y	Y	RRT, CLcr<30 mL/min

Year; Author	Sample size	Study design	Country/ Region	Setting	AKI definition	Trough Level (Y/NS)	AUC_{24h} (Y/NS)	Key Exclusion criteria
2014; Burgess [50]	191	Retrospective	USA	Hospital/ICU	RIFLE	Y	NS	RRT, history of AKI
2013; Mizuno [51]	94	Retrospective	Japan	Hospital	2009 Consensus	Y	NS	-
2012; Horey [52]	270	Retrospective	USA	Hospital	2009 Consensus/RIFLE	Y	NS	receiving concomitant nephrotoxins
2009; Lodise [53]	166	Retrospective	USA	Hospital/ICU	2009 Consensus	Y	Y	cystic fibrosis, vasopressor
2006; Hidayat [54]	95	Prospective	USA	Hospital/ICU	2009 Consensus	Y	NS	-
2020; Flannery [55]	66	Retrospective	USA	ICU	KDIGO	Y	Y	RRT, history of AKI
2019; Hirai [56]	243	Retrospective	Japan	Hospital/ICU	2009 Consensus	Y	NS	RRT
2019; Okada [57]	150	Retrospective	Japan	Hospital	KDIGO	Y	NS	RRT, eGFR <30 mL/min/1.73m^2
2013; Ramírez [58]	179	Prospective	Spain	Hospital/ICU	RIFLE	Y	NS	-
2020; Molina [59]	394	Retrospective	USA	ICU	AKIN	Y	NS	RRT
2019; Sharma [60]	1130	Retrospective	USA	Hospital	AKIN/KDIGO	Y	NS	RRT, CLcr<30 mL/min
2019; Hays [61]	50	Retrospective	USA	ICU	2009 Consensus	Y	NS	RRT, cystic fibrosis, Scr > 1.5 mg/dL, BMI<=15 kg/m^2 or >=40 kg/m^2
2019; Covvey [62]	119	Retrospective	USA	Hospital/ICU	KDIGO	Y	Y	RRT, CLcr<30 mL/min
1995; Zimmermann [63]	45	Retrospective	USA	Hospital/ICU	2009 Consensus	Y	NS	Scr > 3.0 mg/dL, receiving concomitant nephrotoxins
2014; Brumer [64]	100	Retrospective	USA	Hospital/ICU	2009 Consensus	Y	NS	renal insufficiency
2017; Brown [65]	162	Retrospective	USA	Hospital/ICU	2009 Consensus	Y	NS	RRT, CLcr<25 mL/min, cystic fibrosis
2012; Cano [66]	188	Retrospective	USA	ICU	2009 Consensus	Y	NS	RRT, SCr >=2.0 mg/dL, history of AKI
2014; Cappelletty [67]	226	Retrospective	USA	Hospital	2009 Consensus	Y	NS	sepsis or septic shock, history of AKI
2021; Bhasin [68]	280	Retrospective	USA	Hospital/ICU	KDIGO	Y	NS	-
2018; Huang [69]	50	Retrospective	China	ICU	2009 Consensus/AKIN	Y	Y	RRT
2021; Higashi [70]	107	Retrospective	Japan	Hospital	2009 Consensus	Y	NS	-
2016; Fodero [71]	453	Retrospective	USA	Hospital/ICU	2009 Consensus	Y	NS	RRT
2013; Golenia [72]	117	Retrospective Prospective	USA	ICU	2009 Consensus	Y	NS	RRT, eGFR <=30 mL/min/1.73m^2
2017; Hale [73]	100	Retrospective	USA	Hospital/ICU	2009 Consensus	Y	Y	RRT
2021; Hall [74]	84	Retrospective	USA	Hospital	AKIN	Y	NS	RRT, decompensated heart failure
2014; Moh'd [75]	579	Retrospective	USA	Hospital/ICU	2009 Consensus	Y	NS	RRT
2014; Han [76]	1269	Retrospective	Korea	Hospital/ICU	2009 Consensus	Y	NS	impaired kidney function, baseline serum creatinine >=2 mg/dL, dosing interval more than 24 hours
2020; Haruki [77]	272	Retrospective	Japan	Hospital/ICU	RIFLE	Y	NS	RRT, SCr >=1.2 mg/dL

Year; Author	Sample size	Study design	Country/ Region	Setting	AKI definition	Trough Level (Y/NS)	AUC_{24h} (Y/NS)	Key Exclusion criteria
2010; Hermsen [78]	55	Retrospective	USA	Hospital/ICU	2009 Consensus	Y	NS	CLcr<=30 mL/min
2015; Hong [79]	130	Retrospective	USA	ICU	2009 Consensus	Y	Y	RRT, CLcr<20 mL/min
2013; Ghehi [80]	46	Prospective	Iran	Hospital	AKIN	Y	NS	discontinued prior to achieving a steady state
2017; Imai [81]	592	Retrospective	Japan	Hospital/ICU	2009 Consensus	Y	NS	RRT, history of AKI
2019; Imai [82]	402	Retrospective	Japan	Hospital/ICU	2009 Consensus	Y	NS	RRT, history of AKI
2016; Ko [83]	378	Retrospective	USA	Trauma	AKIN/KDIGO	Y	NS	
2011; Kullar [84]	200	Prospective	USA	Hospital/ICU	2009 Consensus	Y	NS	Weight>110 kg, CLcr<30 mL/min, CLcr>110 mL/min, receiving concurrent vasopressors, SCr <0.6 mg/dL
2012; Kullar [85]	200	Retrospective	USA	Trauma	2009 Consensus	Y	NS	RRT
2013; Ley [86]	263	Retrospective	USA	Trauma ICU	2009 Consensus	Y	NS	RRT
2015; Liu [87]	124	Retrospective	China	Hospital/ICU	AKIN	Y	NS	history of AKI, cystic fibrosis, received contrast dye
2015; Masuda [88]	610	Retrospective	Japan	Hospital	2009 Consensus	Y	NS	RRT
2019; Meng [89]	296	Prospective	USA	Hospital	2009 Consensus	Y	Y	RRT, history of AKI
2020; Muklewicz [90]	636	Retrospective	USA	Hospital/ICU	2009 Consensus/AKIN/RIFLE	Y	Y	RRT, change in SCr >0.3 mg/dL
2020; Oda [91]	74	Retrospective	Japan	Hospital/ICU	AKIN/RIFLE	Y	Y	RRT
2018; Park [92]	315	Retrospective	Korea	Hospital/ICU	2009 Consensus	Y	NS	SCr >2.0 mg/dL, RRT, history of AKI
2012; Prabaker [93]	348	Prospective	USA	Hospital	2009 Consensus	Y	NS	SCr >2.0 mg/dL, history of AKI, concomitant amphotericin B
2010; Pritchard [94]	130	Retrospective	USA	Hospital	2009 Consensus	Y	NS	RRT, SCr >3.0 mg/dL
2017; Qian [95]	136	Retrospective	China	ICU	2009 Consensus	Y	NS	renal dysfunction
2012; Reynolds [96]	138	Retrospective	USA	Hospital/ICU	2009 Consensus	Y	NS	CLcr<60 mL/min, unstable renal function
2018; Robertson [97]	169	Retrospective	USA	Hospital	2009 Consensus	Y	NS	Critically ill, CLcr<30 mL/min, RRT
1990; Rybak [98]	231	Prospective	USA	Hospital	2009 Consensus	Y	NS	RRT, septic shock
2020; Sazanami [99]	61	Retrospective	Japan	ICU	KDIGO	Y	NS	RRT
2014; Suzuki [9]	63	Retrospective	Japan	Hospital	2009 Consensus	Y	NS	RRT, other anti-MRSA agents, Gram-negative bacteremia
2021; Alosaimy [100]	154	Retrospective	USA	Hospital/ICU	2009 Consensus	Y	Y	RRT

RCT: randomized controlled trial; PC: prospective cohort; RC: retrospective cohort; ICU: intensive care unit; KDIGO: Kidney Disease Improving Global Outcomes; AKIN: Acute Kidney Injury Network; RIFLE: Risk, Injury, Failure, Loss of kidney function and End-stage kidney disease; RRT: renal replacement therapy; MRSA: methicillin-resistant Staphylococcus aureus; eGFR: estimated glomerular filtration rate; AKI: acute kidney injury; BMI: body mass index; ECMO: extracorporeal membrane oxygenation

Table S2. Patients' characteristics in included studies for Nephrotoxicity

Year; Author	Male gender (%)	Mean age (years)	Mean weight (kg)	Mean CrCL (mL/min)	Mean serum creatinine (mg/dL)	ICU (%)	RRT (%)	Diabetes (%)	Hypertension (%)	PTZ (%)	NSAID (%)	Aminoglycoside (%)	Amphotericin B (%)	ACEi/ ARB (%)	Diuretics (%)	Vasopressors (%)	IV Contrast (%)
2021; Sohn [1]	59.5	60.5	-	-	-	37.8	-	35.1	40.5	-	-	-	-	-	-	-	-
2021; Marko [2]	55.0	62.0	81.2	-	1.09	35.9	0	-	-	-	-	-	-	-	-	28.1	23.4
2021; Katip [3]	43.6	61.2	56.0	38.8	2.0	-	0	15.4	-	14.1	-	1.3	3.2	-	34.9	-	-
2019; Al-Sulaiti [4]	80.0	42.0	71.7	-	0.75	47.7	0	21.5	27.7	-	7.7	-	3.1	-	15.4	27.7	-
2018; Wan [5]	53.4	63.3	-	-	0.90	12.6	0	16.5	-	-	-	-	-	-	-	-	-
2018; Mogle [6]	54.3	50.0	78.4	102	-	41.3	0	30.4	-	67.4	52.2	4.3	-	8.7	23.9	19.6	41.3
2018; Jumah [7]	54.4	75.0	-	48.3	-	14.0	0	36.8	-	-	-	35.1	-	-	-	-	-
2016; Fukumori [8]	58.0	79.4	47.7	64.0	1.12	-	0	-	-	-	-	-	-	-	-	-	-
2015; Suzuki [9]	68.3	50.9	55.6	113.1	0.65	-	0	-	-	4.8	31.7	6.3	6.3	-	52.4	-	-
2020; Zhang [10]	22.8	65.0	-	99.0	0.78	100	-	-	-	-	-	-	-	-	-	-	-
2020; Perin [11]	66.0	67.0	74.0	62.0	0.77	100	27.4	19.0	30.7	-	-	-	-	-	-	-	-
2019; Yahav [12]	62.1	67.4	-	-	1.80	39.9	-	-	-	-	-	-	-	-	-	-	-
2017; Frazee [13]	61.7	64.9	-	98.0	0.90	100	0	23.8	-	-	-	-	-	-	-	-	-
2012; Suzuki [14]	90.3	73.0	52.8	61.8	1.20	-	0	-	-	-	-	-	-	-	-	-	-
2001; Wysocki [15]	64.7	63.0	71.0	73.0	1.05	100	7.6	12.6	-	-	-	18.5	-	-	32.8	-	-
2013; Gawronski [16]	59.3	54.0	-	-	-	27.1	0	-	-	-	-	-	-	-	-	-	-
2016; Duszynska [17]	83.3	58.0	80.5	98.0	1.05	100	0	-	-	-	21.4	4.8	-	-	26.2	-	11.9
2013; Mizokami [18]	67.0	82.6	42.9	39.0	0.73	-	-	-	-	-	-	-	-	-	-	-	-
2017; Zasowski [19]	51.7	61.7	-	-	0.92	-	0	35.9	75.9	0	-	7.7	-	-	41.5	-	18.0
2017; Finch [20]	55.5	59.1	66.1	82.4	0.92	-	0	39.3	73.5	0	-	6.6	-	31.7	38.8	-	10.9
2022; Wang [21]	82.8	57.0	-	91.7	0.96	100	0	-	-	0	7.8	7.8	-	43.8	0	-	9.4
2022; Ueda [22]	65.0	-	-	-	-	17.3	0	-	-	20.8	21.2	0	-	14.2	10.0	-	13.8
2021; Yasu [23]	63.9	44.0	58.9	129	0.60	-	-	-	-	-	-	-	13.9	-	-	-	-
2021; Wang [24]	71.3	88.0	61.0	-	0.67	-	0	37.0	71.1	-	1.1	5.0	-	10.0	22.1	-	-
2021; Liu [25]	62.0	56.4	65.2	101	0.70	31.9	0	11.3	30.1	10.5	29.9	2.8	-	18.0	27.9	16.2	23.4
2021; Johnston [26]	59.6	55.0	79.0	102	0.90	36.0	0	-	-	-	-	-	-	-	-	11.2	-
2021; Al Sulaiman [27]	70.3	58.5	72.3	-	1.20	100	0	50.5	53.7	62.4	-	-	-	-	53.8	-	35.7
2020; Zhang [28]	70.8	60.2	63.7	62.8	1.28	-	0	23.8	47.6	10.7	8.3	-	5.4	4.8	19.6	-	3.6
2020; Ueki [29]	64.2	67.1	55.0	80.7	0.81	50.0	0	23.6	35.1	-	-	-	-	-	-	-	-
2020; Qin [30]	63.1	78.9	-	-	1.03	-	-	27.4	50.7	62.7	15.6	46.0	2.2	40.4	65.2	-	-
2020; Pan [31]	70.1	83.0	-	-	0.86	23.0	0	25.5	-	24.5	-	6.9	2.0	18.1	64.7	23.0	2.9
2020; Mcgrady [32]	53.8	55.7	88.1	-	1.10	0	0	-	-	-	-	-	-	-	-	-	-
2020; Ma [33]	61.0	57.0	84.0	-	-	-	0	20.2	-	12.7	53.1	1.4	-	29.1	27.4	-	28.8
2020; Imai [34]	63.8	65.0	57.0	85.9	0.67	12.7	0	-	-	16.5	47.4	2.3	1.8	-	34.4	13.1	-
2020; Brunetti [35]	67.4	57.0	-	81.2	-	-	0	-	-	-	-	-	-	-	-	-	-
2018; Truong [36]	47.0	58.3	81.1	44.3	1.05	100	0	-	-	-	-	-	-	-	-	-	-
2019; Yahav [37]	62.1	67.0	-	-	1.46	0	-	-	-	-	-	-	-	-	-	-	-
2019; de Almeida [38]	59.2	55.9	70.0	-	0.70	0	0	-	-	15.3	-	-	4.1	-	20.4	-	-
2018; Nakashima [39]	57.9	54.3	56.4	-	0.65	-	0	-	-	-	-	-	-	-	-	-	-
2018; May [40]	69.1	41.6	94.9	144	0.79	100	0	-	-	76.4	10.9	1.8	-	-	38.8	26.7	84.8
2018; Liang [41]	67.6	61.0	-	88.2	0.79	54.0	-	-	-	-	-	-	-	-	-	-	-
2017; Han [42]	57.3	58.8	-	-	-	41.0	20.0	-	-	-	-	-	-	-	-	-	-
2017; Chavada [43]	69.3	-	90.7	-	-	26.0	0	33.9	-	-	-	-	-	-	-	-	-
2017; Anderson [44]	-	58.0	87.0	115	0.90	0	0	-	-	44.4	27.0	4.0	-	28.0	21.0	-	36.0
2016; Hammoud [45]	45.2	56.0	-	-	0.87	-	0	24.9	45.5	-	4.4	1.4	0.0	18.2	-	-	38.4
2015; Hanrahan [46]	65.8	57.0	-	-	-	100	0	13.3	32.9	-	3.2	39.2	5.1	13.3	-	68.4	22.8
2015; Dong [47]	65.6	46.3	62.4	94.8	0.89	100	0	-	-	-	-	0.0	0.0	-	-	0.0	0.0
2014; Hanrahan [48]	65.4	58.3	76.0	-	-	100	-	-	-	-	-	-	-	-	-	-	-
2014; Hall [49]	90.2	74.9	76.1	58.9	1.06	-	0	-	-	-	-	-	-	-	-	-	-
2014; Burgess [50]	53.4	58.4	-	-	-	17.3	-	-	-	-	-	-	-	-	-	-	-

Year; Author	Male gender (%)	Mean age (years)	Mean weight (kg)	Mean CrCL (mL/min)	Mean serum creatinine (mg/dL)	ICU (%)	RRT (%)	Diabetes (%)	Hypertension (%)	PTZ (%)	NSAID (%)	Aminoglycoside (%)	Amphotericin B (%)	ACEi/ ARB (%)	Diuretics (%)	Vasopressors (%)	IV Contrast (%)
2013; Mizuno [51]	67.0	82.6	42.9	39.0	0.72	-	-	-	-	-	-	-	-	-	-	-	-
2012; Horey [52]	96.7	67.4	89.9	66.8	1.20	-	-	51.5	-	54.4	11.1	0.0	0.0	27.0	44.8	0.0	0.0
2009; Lodise [53]	53.6	55.8	80.0	72.4	0.80	42.2	-	31.3	-	-	-	10.2	-	-	-	0.9	0.0
2006; Hidayat [54]	41.1	72.5	-	-	1.02	42.1	-	41.1	-	-	-	-	-	-	-	-	-
2020; Flannery [55]	53.0	49.9	82.4	109	0.90	100	0	-	-	-	-	-	-	-	-	-	-
2019; Hirai [56]	68.3	71.1	55.1	80.0	0.83	33.3	0	-	-	-	20.6	-	-	18.9	22.2	-	
2019; Okada [57]	56.0	56.6	57.4	111	-	-	0	-	-	15.3	10.0	-	7.3	9.3	13.3	0.7	-
2013; Ramírez [58]	45.3	66.3	79.6	92.4	0.83	34.6	-	-	-	-	-	-	-	-	-	-	-
2020; Molina [59]	65.7	54.0	88.3	118	0.77	100	0	17.8	22.3	65.5	12.9	9.1	0.8	31.5	68.8	42.1	22.1
2019; Sharma [60]	58.8	52.7	-	-	0.80	-	0	29.6	-	-	30.4	3.4	0.1	28.9	29.1	-	20.1
2019; Hays [61]	62.0	19.9	68.6	-	0.59	100	0	-	-	-	-	-	-	-	-	36.0	-
2019; Covvey [62]	57.1	59.4	88.9	78.0	1.20	47.1	0	-	-	-	21.0	-	-	18.5	47.1	-	44.5
1995; Zimmermann [63]	55.6	60.0	-	68.4	0.90	8.9	-	11.1	17.8	0.0	0.0	0.0	0.0	0.0	0.0	0.0	0.0
2014; Brumer [64]	53.0	38.0	67.0	124	-	23.0	-	4.0	-	-	24.0	26.0	-	12.0	29.0	7.0	59.0
2017; Brown [65]	56.8	57.5	90.6	82.4	1.10	27.8	0	30.9	-	43.8	20.4	4.3	-	32.7	37.7	13.6	16.0
2012; Cano [66]	62.8	58.5	84.4	-	-	100	0	27.7	-	-	-	33.0	-	-	-	-	-
2014; Cappelletty [67]	56.2	58.7	-	71.6	-	-	-	36.7	57.5	-	-	-	-	32.3	47.8	-	28.8
2021; Bhasin [68]	55.0	589.0	-	-	0.87		-	-	-	-	-	-	-	-	-	-	-
2018; Huang [69]	88.0	85.0	-	56.5	0.83	100	0	34.0	72.0	-	6.0	-	0.0	14.0	88.0	28.0	-
2021; Higashi [70]	69.2	57.8	61.9	91.9	0.60	-	-	20.6	-	8.4	65.4	3.7	5.6	-	12.1	6.5	45.8
2016; Fodero [71]	97.4	68.2	89.6	73.3	1.07	21.2	0	41.3	-	63.6	10.4	1.3	-	31.6	44.2	-	-
2013; Golenia [72]	63.2	60.5	76.0	83.1	-	100	0	-	-	-	-	-	-	-	-	-	-
2017; Hale [73]	59.0	59.2	77.7	102	0.70	14.0	0	-	-	-	-	-	-	-	-	-	-
2021; Hall [74]	60.7	55.8	79.8	98.8	0.98	-	0	-	-	47.6	-	3.6	-	-	29.8	-	2.4
2014; Moh'd [75]	58.2	55.3	89.9	100	0.88	24.2	0	28.2	47.3	-	-	3.8	-	-	18.0	-	30.2
2014; Han [76]	62.3	57.6	59.9	-	1.00	35.9	-	-	-	-	-	-	-	-	-	-	-
2020; Haruki [77]	61.0	75.8	53.5	62.9	0.73	27.2	0	16.2	-	-	-	-	-	-	-	-	-
2010; Hermsen [78]	60.0	58.8	-	-	-	18.2	-	-	-	-	-	-	-	-	-	-	-
2015; Hong [79]	54.6	56.2	81.1	89.3	0.95	100	0	-	-	-	-	-	-	-	-	-	-
2013; Ghehi [80]	65.2	32.9	74.8	-	-	-	-	-	-	-	-	-	-	-	-	-	-
2017; Imai [81]	64.5	60.8	58.2	91.2	0.74	12.5	0	-	-	-	44.6	2.7	0.8	-	30.6	11.1	-
2019; Imai [82]	63.2	62.5	56.8	-	0.71	7.2	0	-	-	12.9	50.0	1.0	0.5	-	25.4	8.7	-
2016; Ko [83]	79.9	48.6	-	146	0.80	-	2.4	10.6	-	-	14.0	19.3	-	-	39.7	24.3	78.0
2011; Kullar [84]	62.0	56.0	71.2	66.5	-	26.0	-	-	-	-	-	-	-	-	-	0.0	-
2012; Kullar [85]	-	53.8	72.5	66.8	-	-	-	-	-	-	-	-	-	-	-	-	-
2013; Ley [86]	80.6	50.0	-	-	0.80	-	0	-	-	-	-	-	-	-	-	-	-
2015; Liu [87]	67.7	67.8	-	99.3	0.94	37.9	-	42.7	29.8	-	-	-	-	64.5	23.4	-	0
2015; Masuda [88]	68.2	73.2	53.8	73.1	0.74	-	0	-	-	-	-	-	-	-	-	-	-
2019; Meng [89]	55.7	57.7	79.2	-	0.79	-	0	-	-	-	-	-	-	-	-	-	-
2020; Muklewicz [90]	58.8	59.7	85.5	-	0.90	37.9	0	36.2	-	37.1	13.2	1.3	-	28.6	43.2	-	57.9
2020; Oda [91]	56.8	59.6	58.4	-	-	18.9	0	37.8	50.0	-	17.6	-	-	-	17.6	-	-
2018; Park [92]	66.7	58.0	-	-	0.68	42.2	0	-	-	-	-	5.4	-	9.5	34.0	-	-
2012; Prabaker [93]	97.4	60	-	-	1.13	-	-	35.9	-	-	10.6	19.0	0	8.6	25.0	5.7	12.6
2010; Pritchard [94]	-	61.9	88.7	-	1.02	-	0	-	-	-	-	-	-	-	-	-	-
2017; Qian [95]	61.8	62.9	-	-	0.80	100	-	-	-	-	-	-	-	-	-	-	-
2012; Reynolds [96]	54.3	53.6	123	126	0.80	22.5		41.3	-	-	-	-	-	-	-	-	-
2018; Robertson [97]	54.4	57.9	87.5	-	0.79	0	0	34.9	50.3	50.3	36.1	0	-	21.3	28.4	-	55.0
1990; Rybak [98]	66.7	39.3	65.8	95.1	-	-	0	-	-	-	-	-	0.0	-	0.0	-	-
2020; Sazanami [99]	68.9	69.0	52.0	55.2	0.70	100	0	-	-	24.6	3.3	-	-	9.8	6.6	-	-
2014; Suzuki [9]	68.3	50.9	55.6	113	0.65	-	-	-	-	-	31.7	-	-	-	52.4	-	-
2021; Alosaimy [100]	64.3	50.5	82.0	-	1.00	14.3	0	-	-	25.9	5.2	2.6	-	14.3	-	-	5.8

CrCL: creatinine clearance; ACEi: angiotensin converting enzyme inhibitor; ARB: angiotensin receptor blocker; PTZ: piperacillin-tazobactam; NSAID: nonsteroidal anti-inflammatory drug.

Table S3. Methodological characteristics of the included studies for Efficacy

Year; Author	Sample size	Study design	Type of infection	MRSA (%)	Country/ Region	Setting	Trough Level	AUC_{24h}/ MIC	MIC method	Outcome	Outcome type (Proportion and/or Binary)
2022; Ueda [22]	260	Retrospective	Bacteremia, pneumonia, mixed	100	Japan	Hospital/ICU	Y	NS	BMD	All-cause mortality; Clinical failure	Proportion
2022; Fan [101]	63	Prospective	Pneumonia	100	China	Hospital/ICU	Y	Y	Agar dilution	Clinical failure; Microbiological failure	Proportion and Binary
2021; Wang [24]	349	Retrospective	Bacteremia, pneumonia, mixed	12.3	China	Hospital	Y	NS	NS	Treatment failure; All-cause mortality; Clinical failure; Microbiological failure	Proportion and Binary
2021; Ren [102]	7220	Retrospective	Pneumonia, mixed	-	China	ICU	Y	NS	NS	All-cause mortality	Proportion
2021; Marko [2]	128	Retrospective	Bacteremia, pneumonia	100	Canada	Hospital/ICU	Y	Y	Agar dilution	Treatment failure; All-cause mortality; Bacteremia recurrence; Microbiological failure	Proportion and Binary
2021; Lines [6]	156	Retrospective	Bacteremia	100	USA	Hospital/ICU	Y	NS	VITEK 2	Treatment failure; All-cause mortality; Clinical failure	Proportion
2021; Hou [103]	3603	Retrospective	NS	-	China	ICU	Y	NS	NS	All-cause mortality	Proportion
2021; Alosaimy [100]	154	Retrospective	cSSTIs, Bacteremia	100	USA	Hospital/ICU	Y	NS	NS	All-cause mortality; Clinical failure	Proportion and Binary
2021; Al Sulaiman [27]	210	Retrospective	Bacteremia, pneumonia, mixed	-	Saudi Arabia	ICU	Y	NS	NS	All-cause mortality	Proportion
2021; Katip [3]	312	Retrospective	Bacteremia, mixed	0	Thailand	Hospital	Y	Y	VITEK 2	Clinical failure; Microbiological failure	Proportion
2020; Zhang [10]	57	Retrospective	Pneumonia	28.1	China	ICU	Y	NS	NS	All-cause mortality; Clinical failure	Proportion
2020; Perin [11]	179	Retrospective	Bacteremia, pneumonia, mixed	4.9	France	ICU	Y	NS	NS	All-cause mortality; Clinical failure	Proportion
2020; Oda [91]	74	Retrospective	Pneumonia, mixed	36.5	Japan	Hospital/ICU	Y	NS	NS	All-cause mortality	Proportion
2020; Lodise [104]	265	Prospective	Bacteremia	100	USA	Hospital/ICU	Y	Y	BMD; Etest	Treatment failure; All-cause mortality; Bacteremia recurrence; Microbiological failure	Proportion
2020; Chattaweelarp [105]	131	Retrospective	Bacteremia, pneumonia, mixed	100	Thailand	Hospital/ICU	NS	Y	BMD	Treatment failure; All-cause mortality; Microbiological failure	Proportion
2019; Yahav [12]	285	Retrospective	Bacteremia, pneumonia, mixed	100	Israel	Hospital/ICU	Y	Y	Etest	All-cause mortality; Clinical failure; Microbiological failure	Proportion

Year; Author	Sample size	Study design	Type of infection	MRSA (%)	Country/ Region	Setting	Trough Level	AUC_{24h}/ MIC	MIC method	Outcome	Outcome type (Proportion and/or Binary)
2019; Yahav [37]	285	Retrospective	Bacteremia, pneumonia, mixed	100	Israel	Hospital	Y	NS	NS	All-cause mortality; Clinical failure; Microbiological failure	Proportion
2021; Johnston [26]	89	Retrospective	Bacteremia	100	USA	Hospital/ICU	Y	NS	NS	Treatment failure; All-cause mortality; Bacteremia recurrence; Microbiological failure	Proportion and Binary
2019; Makmor-Bakry [106]	28	Retrospective	Bacteremia	100	Malaysia	ICU	Y	Y	Y	All-cause mortality; Clinical failure	Proportion and Binary
2019; de Almeida [38]	98	Prospective	Bacteremia, mixed	6.1	Brazil	Hospital	Y	NS	NS	All-cause mortality	Proportion
2019; Clark [107]	34	Retrospective	Bacteremia, pneumonia, mixed	-	USA	Hospital/ICU	Y	Y	MicroScan	All-cause mortality	Proportion
2019; Al-Sulaiti [4]	65	RCT	Bacteremia, pneumonia, mixed	26.2	Qatar	Hospital/ICU	Y	Y	BMD	Treatment failure; All-cause mortality	Proportion
2018; Wan [5]	103	Retrospective	Bacteremia, mixed	9.7	Canada	Hospital/ICU	Y	NS	NS	All-cause mortality; Clinical failure; Bacteremia recurrence	Proportion
2018; Shen [108]	334	Prospective	Bacteremia, pneumonia	-	China	Hospital	Y	Y	Agar dilution	Clinical failure; Microbiological failure	Proportion and Binary
2018; Liang [41]	370	Prospective	Bacteremia, pneumonia, mixed	-	China	Hospital/ICU	Y	NS	Agar dilution	Treatment failure	Proportion and Binary
2018; Komoto [109]	77	Retrospective	Bacteremia	100	Japan	Hospital	Y	NS	MicroScan	All-cause mortality	Proportion
2018; Jumah [7]	57	Retrospective	Bacteremia	0	Singapore	Hospital/ICU	Y	Y	Etest	All-cause mortality	Proportion and Binary
2018; Huang [69]	50	Retrospective	Bacteremia, pneumonia, mixed	24.0	China	ICU	Y	NS	NS	All-cause mortality; Clinical failure; Microbiological failure	Proportion and Binary
2018; Fu [110]	42	Retrospective	Bacteremia	100	China	Hospital	Y	NS	BMD	Treatment failure; All-cause mortality; Bacteremia recurrence; Microbiological failure	Proportion and Binary
2017; Frazee [13]	399	Prospective	Mixed	9.0	USA	ICU	Y	NS	NS	All-cause mortality	Proportion
2016; Moise [111]	85	Retrospective	Bacteremia	100	USA	Hospital/ICU	Y	NS	NS	Treatment failure; All-cause mortality	Proportion
2016; Ko [83]	378	Retrospective	Pneumonia, mixed	-	USA	Trauma	Y	NS	NS	All-cause mortality (NS)	Proportion
2016; Ji [112]	47	Retrospective	Pneumonia	100	Korea	Hospital	Y	Y	MicroScan	Microbiological failure	Proportion and Binary
2016; Fukumori [8]	81	Retrospective	Pneumonia	100	Japan	Hospital	Y	NS	NS	Treatment failure	Proportion and Binary
2016; Duszynska [17]	42	Prospective	Mixed	0	Poland	ICU	NS	Y	Etest	Clinical failure; Microbiological failure	Proportion and Binary

Year; Author	Sample size	Study design	Type of infection	MRSA (%)	Country/ Region	Setting	Trough Level	AUC_{24h}/ MIC	MIC method	Outcome	Outcome type (Proportion and/or Binary)
2015; Suzuki [9]	63	Retrospective	Bacteremia, pneumonia, mixed	-	Japan	Hospital	Y	NS	NS	Clinical failure	Proportion and Binary
2015; Stevenson [113]	34	Retrospective	Mixed	-	Australia	Hospital	Y	NS	NS	Treatment failure	Proportion and Binary
2015; Song [114]	117	Prospective	Bacteremia	100	Korea	Hospital	Y	Y	BMD; Etest	Treatment failure; All-cause mortality (30-day); Bacteremia recurrence; Microbiological failure	Proportion and Binary
2015; Casapao [115]	139	Retrospective	Endocarditis	100	USA	Hospital/ICU	Y	Y	BMD; Etest	Treatment failure; Microbiological failure	Proportion and Binary
2015; Cao [116]	111	Retrospective	Mixed	79.6	China	Hospital	Y	NS	Agar dilution	Treatment failure; Microbiological failure	Proportion and Binary
2014; Lodise [117]	123	Retrospective	Bacteremia	100	USA	Hospital/ICU	Y	Y	BMD; Etest	Treatment failure; All-cause mortality (30-day); Bacteremia recurrence; Microbiological failure	Proportion
2014; Lin [118]	66	Retrospective	Bacteremia, pneumonia, mixed	-	China	ICU	Y	NS	NS	All-cause mortality (28-day)	Proportion
2014; Jung [119]	76	Retrospective	Bacteremia	100	Korea	Hospital	Y	Y	BMD; Etest	Treatment failure; All-cause mortality (30-day); Bacteremia recurrence; Microbiological failure	Proportion and Binary
2014; Hall [49]	92	Retrospective	Bacteremia	100	USA	Hospital/ICU	Y	NS	NS	All-cause mortality (NS)	Proportion
2014; Ghosh [120]	127	Retrospective	Bacteremia	100	Australia	Hospital/ICU	Y	Y	BMD; Etest	Treatment failure; All-cause mortality (30-day); Microbiological failure	Proportion and Binary
2013; Zelenitsky [121]	35	Retrospective	Septic Shock	100	Canada	ICU	Y	Y	BMD	All-cause mortality (NS)	Proportion and Binary
2013; Mizokami [18]	94	Retrospective	Pneumonia	100	Japan	Hospital	Y	NS	MicroScan	All-cause mortality (28-day)	Proportion and Binary
2013; Ley [86]	263	Retrospective	Mixed	-	USA	Trauma ICU	Y	NS	NS	All-cause mortality (NS)	Proportion
2013; Holmes [122]	182	Retrospective	Bacteremia	100	Australia	Hospital/ICU	Y	Y	BMD; Etest	All-cause mortality (30-day)	Proportion and Binary
2013; Gawronski [16]	59	Retrospective	Bacteremia, Osteomyelitis	100	USA	Hospital/ICU	Y	Y	Etest	All-cause mortality (NS); Microbiological failure	Proportion
2012; Suzuki [14]	31	Retrospective	Pneumonia	100	Japan	Hospital	Y	Y	BMD	Microbiological failure	Proportion and Binary
2012; Moore [123]	118	Retrospective	Bacteremia	100	USA	Hospital/ICU	Y	NS	Etest	Treatment failure; All-cause mortality (60-day); Bacteremia recurrence; Microbiological failure	Proportion and Binary
2012; Cheong [124]	76	Retrospective	Mixed	100	Malaysia	ICU	Y	NS	NS	Clinical failure	Proportion and Binary

Year; Author	Sample size	Study design	Type of infection	MRSA (%)	Country/ Region	Setting	Trough Level	AUC_{24h}/ MIC	MIC method	Outcome	Outcome type (Proportion and/or Binary)
2012; Brown [125]	50	Retrospective	Bacteremia, Endocarditis	100	USA	Hospital/ICU	NS	Y	Etest	All-cause mortality (NS)	Proportion
2011; Clemens [126]	94	Retrospective	Bacteremia	100	USA	Trauma	Y	NS	Etest	Treatment failure; All-cause mortality (30-day); Bacteremia recurrence; Microbiological failure	Proportion
2011; Chung [127]	68	Prospective	Pneumonia	100	Korea	ICU	Y	NS	NS	Clinical failure	Proportion
2011; Chan [128]	72	Retrospective	Pneumonia	100	USA	Trauma	Y	NS	NS	Treatment failure; All-cause mortality (NS)	Proportion
2011; Kullar [129]	320	Retrospective	Bacteremia	100	USA	Hospital	Y	Y	BMD	Treatment failure; All-cause mortality (30-day); Clinical failure; Microbiological failure	Proportion and Binary
2010; Hermsen [78]	55	Retrospective	Pneumonia, mixed	100	USA	Hospital/ICU	Y	NS	NS	Treatment failure; All-cause mortality (27 or 36-day)	Proportion
2006; Mohammedi [130]	40	Prospective	Sepsis	45.0	France	ICU	Y	NS	NS	All-cause mortality (NS); Clinical failure	Proportion
2006; Jeffres [131]	102	Retrospective	Pneumonia	100	USA	Hospital	Y	NS	NS	All-cause mortality (NS)	Proportion and Binary
2006; Hidayat [54]	95	Prospective	Bacteremia, pneumonia, mixed	100	USA	Hospital/ICU	Y	NS	Etest	All-cause mortality (NS); Clinical failure	Proportion
2004; Moise-Broder [132]	90	Retrospective	Mixed	36.7	USA	Hospital/ICU	NS	Y	BMD	Treatment failure	Proportion and Binary
2004; Vuagnat [133]	44	Prospective	Osteomyelitis	70.5	France	Hospital	Y	NS	Etest	Clinical failure	Proportion
2001; Wysocki [15]	119	Prospective	Bacteremia, pneumonia, mixed	79.8	France	ICU	Y	NS	Agar dilution	All-cause mortality (EOT); Clinical failure	Proportion and Binary
2000; Moise [134]	56	Retrospective	Pneumonia, mixed	53.6	USA	Hospital/ICU	NS	Y	BMD	Clinical failure	Proportion and Binary
1999; Karam [135]	240	Retrospective, Prospective	Mixed	-	USA	Trauma	Y	NS	NS	Clinical failure	Proportion
2012; Rojas [136]	104	Retrospective	Bacteremia	100	Spain	Hospital/ICU	Y	NS	BMD; Etest	All-cause mortality (NS)	Proportion and Binary
2012; Kullar [85]	200	Retrospective	Bacteremia	100	USA	Trauma	Y	NS	NS	Treatment failure; All-cause mortality (30-day); Clinical failure; Microbiological failure	Proportion
2020; Muklewicz [70]	636	Retrospective	Bacteremia, pneumonia, mixed	-	USA	Hospital/ICU	Y	NS	NS	All-cause mortality (NS)	Proportion
2013; Mizuno [51]	94	Retrospective	Pneumonia	100	Japan	Hospital	Y	Y	NS	All-cause mortality (28-day)	Proportion
2015; Dong [47]	90	Retrospective	Bacteremia, pneumonia,	100	China	ICU	Y	NS	NS	Clinical failure	Proportion

Year; Author	Sample size	Study design	Type of infection	MRSA (%)	Country/ Region	Setting	Trough Level	AUC_{24h}/ MIC	MIC method	Outcome	Outcome type (Proportion and/or Binary)
			mixed								
2017; Chavada [43]	127	Retrospective	Bacteremia	100	Australia	Hospital/ICU	Y	NS	NS	All-cause mortality (30-day)	Proportion
2022; Wang [21]	64	Retrospective	Bacteremia, pneumonia, mixed	-	China	ICU	Y	NS	NS	All-cause mortality (28-day)	Proportion

Mixed infection, urinary tract infection, endocarditis, osteomyelitis, skin and soft tissue, central nervous system infection, catheter-related infection and so on (study not reported bacteremia and pneumonia and specific infection, recorded as mixed). NS, not specified. cSSTIs, complicated skin and soft tissue infections; BMD, broth microdilution method; sample size are the total number of patients in the publication. EOT, end of treatment.

Table S4. Patients' characteristics in included studies for Efficacy

Year; Author	Male gender (%)	Mean age (years)	Mean weight (kg)	Mean CrCL (mL/min)	Mean serum creatinine (mg/dL)	ICU (%)	RRT (%)
2022; Ueda [22]	65.0	-	-	-	-	17.3	0
2022; Fan [101]	69.8	62.0	60.0	-	-	61.9	3.2
2021; Wang [24]	71.3	88.0	61.0	-	0.67	-	0
2021; Ren [102]	56.5	-	-	70.9	1.01	100	2.4
2021; Marko [2]	55.0	62.0	81.2	-	1.09	35.9	0
2021; Lines [137]	-	57.5	-	-	-	24.4	17.9
2021; Hou [103]	58.2	-	-	96.6	0.89	100	6.4
2021; Alosaimy [100]	64.3	50.5	82.0	-	1.00	14.3	0
2021; Al Sulaiman [27]	70.3	58.5	72.3	-	1.20	100	0
2021; Katip [3]	43.6	61.2	56.0	38.8	1.98	-	0
2020; Zhang [10]	22.8	65.0	-	99.0	0.78	100	-
2020; Perin [11]	66.5	67.0	74.0	62.0	0.77	100	27.4
2020; Oda [91]	56.8	59.6	58.4	-	-	18.9	0
2020; Lodise [104]	63.4	60.7	81.7	85.4	<2.0	22.3	0
2020; Chattaweelarp [105]	67.2	70.1	61.5	30.9	-	62.6	32.1
2019; Yahav [12]	62.1	67.4	-	-	1.80	39.9	-
2019; Yahav [37]	62.1	67.0	-	-	1.46	0	-
2021; Johnston [26]	59.6	55.0	79.0	102	0.90	36.0	0
2019; Makmor-Bakry [106]	78.6	59.2	66.4	48.9	-	100	-
2019; de Almeida [38]	59.2	55.9	70.0	-	0.70	0	0
2019; Clark [107]	61.8	63.0	75.0	82.3	0.85	91.2	0
2019; Al-Sulaiti [4]	80.0	40.0	71.7	-	0.75	47.7	0
2018; Wan [5]	53.4	63.3	-	-	0.90	12.6	0
2018; Shen [108]	66.2	61.4	61.7	88.7	0.77	-	-
2018; Liang [41]	67.6	61.0	-	88.2	0.79	54.1	-
2018; Komoto [109]	57.1	76.6	51.1	-	-	-	0
2018; Jumah [7]	54.4	75.0	-	48.3	-	14.0	0
2018; Huang [69]	88.0	85.0	-	56.5	0.83	100	0
2018; Fu [110]	47.6	69.0	56.2	-	-	-	100
2017; Frazee [13]	61.7	64.9	-	98.0	0.90	100	0
2016; Moise [111]	56.5	60.0	70.0	91.0	-	30.6	27.1
2016; Ko [83]	79.9	48.6	-	146.2	0.80	-	2.4
2016; Ji [112]	80.9	67.9	56.5	70.1	-	-	-
2016; Fukumori [8]	58.0	79.4	47.7	64.0	1.12	-	0
2016; Duszynska [17]	83.3	58.0	80.5	98.0	1.05	100	0
2015; Suzuki [9]	68.3	50.9	55.6	113.1	0.65	-	0
2015; Stevenson [113]	67.6	58.2	-	-	-	-	100
2015; Song [114]	70.1	67.0	58.6	-	-	-	0

Year; Author	Male gender (%)	Mean age (years)	Mean weight (kg)	Mean CrCL (mL/min)	Mean serum creatinine (mg/dL)	ICU (%)	RRT (%)
2015; Casapao [115]	66.9	51.0	72.0	62.1	-	45.1	0
2015; Cao [116]	82.0	72.3	-	78.4	0.76	-	-
2014; Lodise [117]	69.1	61.3	84.8	73.9	-	24.4	0
2014; Lin [118]	75.8	56.0	-	-	-	100	0
2014; Jung [119]	76.3	69.0	60.5	-	-	-	0
2014; Hall [49]	90.2	74.9	76.1	58.9	1.06	-	0
2014; Ghosh [120]	68.5	64.6	70.0	67.0	-	26.0	0
2013; Zelenitsky [121]	62.9	61.9	70.9	64.0	-	100	-
2013; Mizokami [18]	67.0	82.6	42.9	39.0	0.73	-	-
2013; Ley [86]	80.6	50.0	-	-	0.80	-	0
2013; Holmes [122]	70.3	-	74.0	-	-	28.5	18.1
2013; Gawronski [16]	59.3	54.0	-	-	-	27.1	0
2012; Suzuki [14]	90.3	73.0	52.8	61.8	1.20	-	0
2012; Moore [123]	62.7	52.0	-	-	-	21.2	0
2012; Cheong [124]	75.0	55.5	68.4	80.1	0.93	100	-
2012; Brown [125]	50.0	54.8	-	-	-	22.0	0
2011; Clemens [126]	78.7	52.3	-	-	-	40.4	9.6
2011; Chung [127]	73.5	61.4	-	61.7	-	100	-
2011; Chan [128]	69.8	53.9	-	-	-	-	-
2011; Kullar [129]	-	53.8	73.1	62.8	-	-	12.8
2010; Hermsen [78]	60.0	58.8	-	-	-	18.2	-
2006; Mohammedi [130]	72.5	61.5	-	-	-	100	-
2006; Jeffres [131]	61.8	59.4	-	-	-	-	-
2006; Hidayat [54]	41.1	72.5	-	-	1.02	42.1	-
2004; Moise-Broder [132]	53.7	70.2	72.7	49.5	-	61.1	-
2004; Vuagnat [133]	-	56.7	-	-	-	-	-
2001; Wysocki [15]	64.7	63.0	71.0	73.0	1.05	100	7.6
2000; Moise [134]	62.5	69.2	-	50.8	-	67.9	-
1999; Karam [135]	64.2	48.1	75.0	81.1	1.03	55.8	-
2012; Rojas [136]	73.1	65.0	-	-	-	20.2	-
2012; Kullar [85]	-	53.8	72.5	66.8	-	-	-
2020; Muklewicz [90]	58.8	59.7	85.5	-	0.90	37.9	0
2013; Mizuno [51]	67.0	82.6	42.9	39.0	0.72	-	-
2015; Dong [47]	65.6	46.3	62.4	94.8	0.89	100	0
2017; Chavada [43]	69.3	-	90.7	-	-	26.0	0
2022; Wang [21]	82.8	57.0	-	91.7	0.96	100	0

The summary is based on the total number of patients in the publication. CrCL: creatinine clearance;ICU: intensive care unit; RRT: renal replacement therapy, including any type of dialysis

2: SUPPLEMENTARY FIGURES

Figure S1. Probability of Nephrotoxicity for the Troughs Category

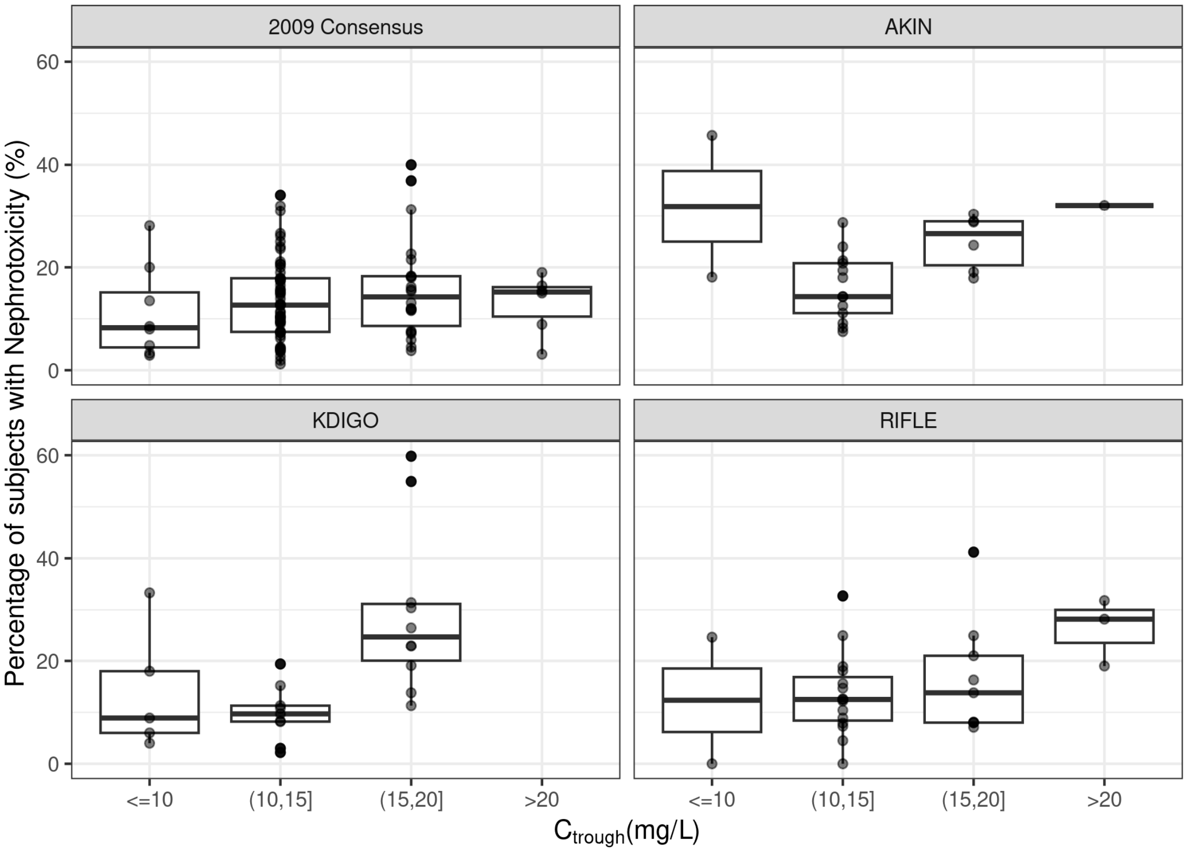

Figure S2. Receiver operating characteristic curve of predictive level of vancomycin trough concentration for nephrotoxicity.

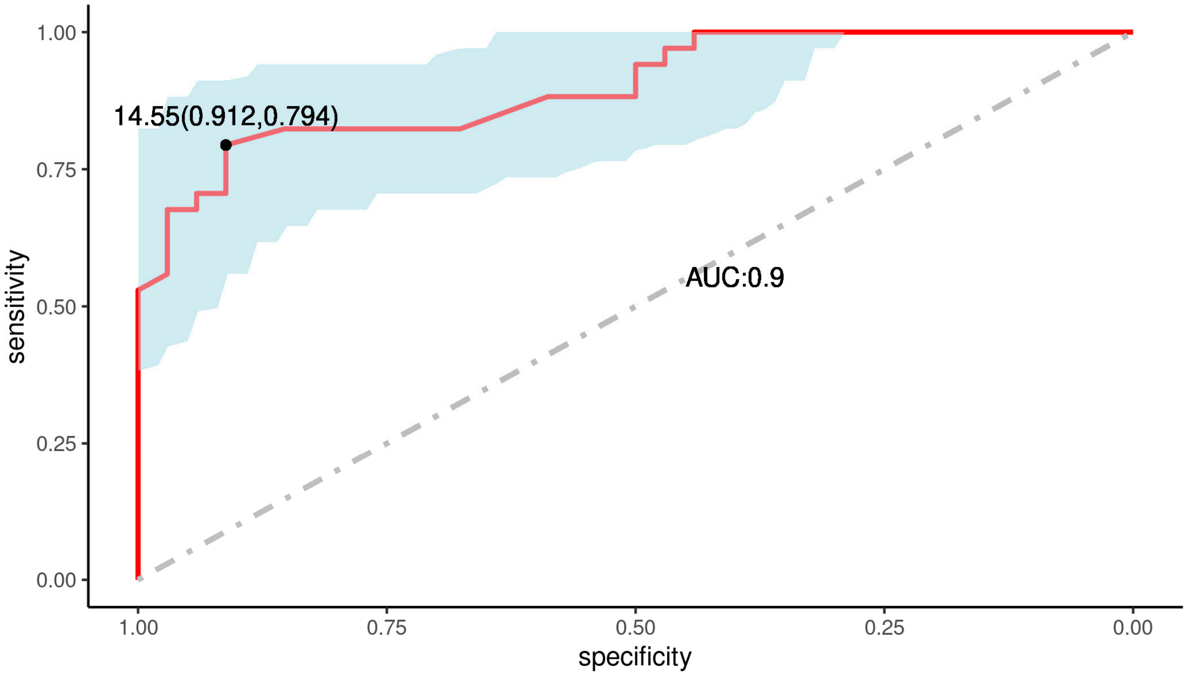

The 14.55 mg/L cut point indicates 79.4% sensitivity and 91.2% specificity on vancomycin-related nephrotoxic adverse effects.

Abbreviations: AUC, area under the receiver operating characteristic curve.

Figure S3. Logistic Regression Illustrating the Association of the Probability of Experiencing Nephrotoxicity as a Function of and Vancomycin Trough Concentrations in patients without any form of dialysis

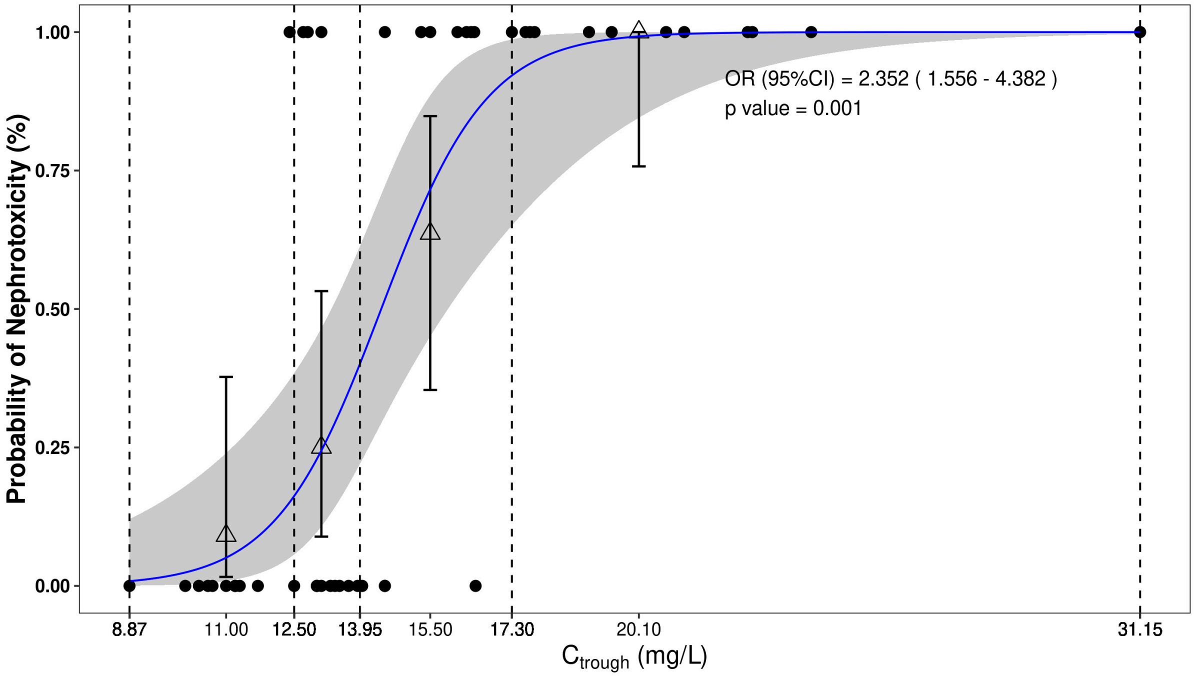

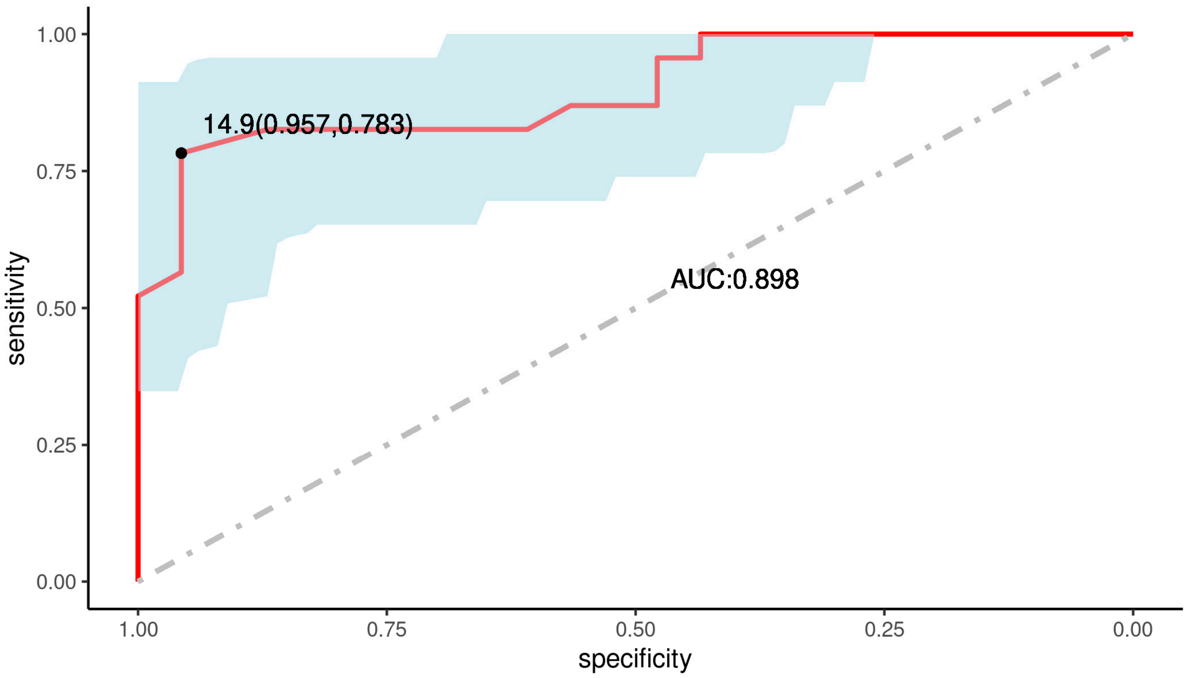

The upper and lower open circles represent the presence or absence of a given Nephrotoxicity across the range of vancomycin trough concentrations, respectively. The dots depict the observed incidence for the quartiles of exposure, whereas the corresponding vertical bars represent the exact 95% CI calculated using Wilson's method. Finally, the middle line and its corresponding shaded area represent model-based exposure-safety relationship and the 95% CI respectively. Vertical dashed lines represent min, 25%, median, 75% and max percentile of trough concentrations, respectively.

Figure S4. Correlation between Nephrotoxicity and AUC_{24}

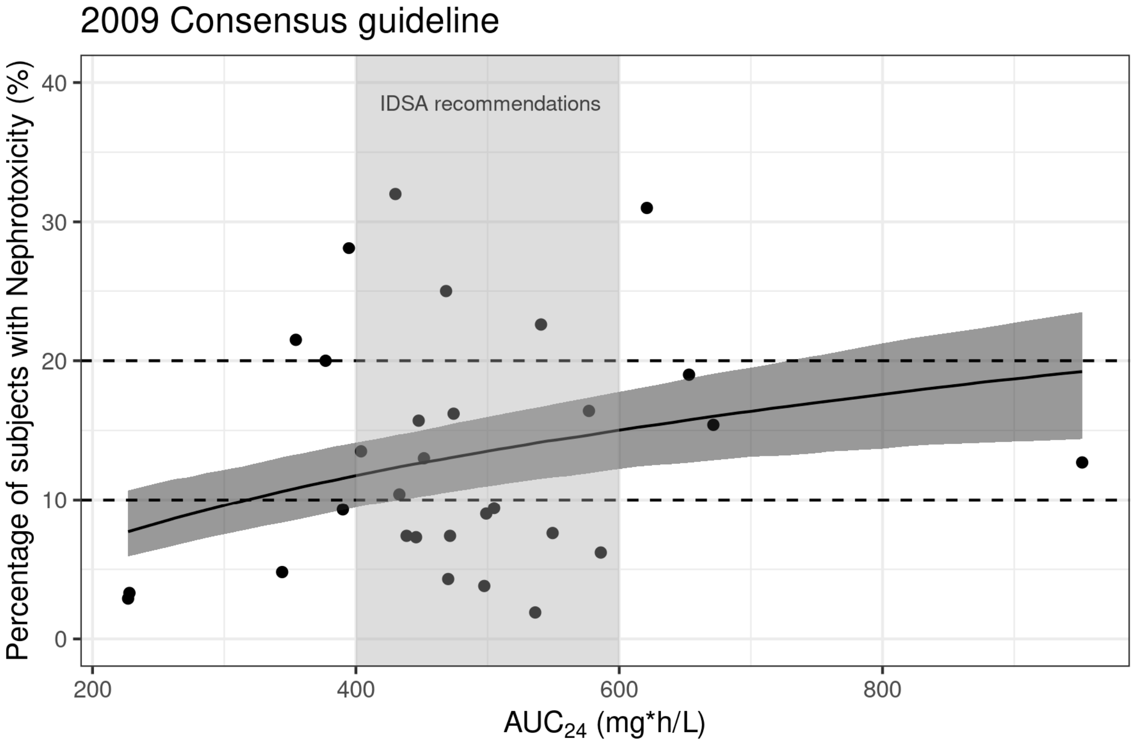

Black circles represent the observations from each study. Solid line and the shaded area represent the estimated Emax model curve with 95% credible intervals of parameters. The light grey shade represents the AUC_{24} interval between 400 mg*h/L and 600 mg*h/L.

Figure S5. Logistic Regression Illustrating the Association of the Treatment Success Rates as a Function of and Vancomycin AUC_{24}/MIC_{BMD}

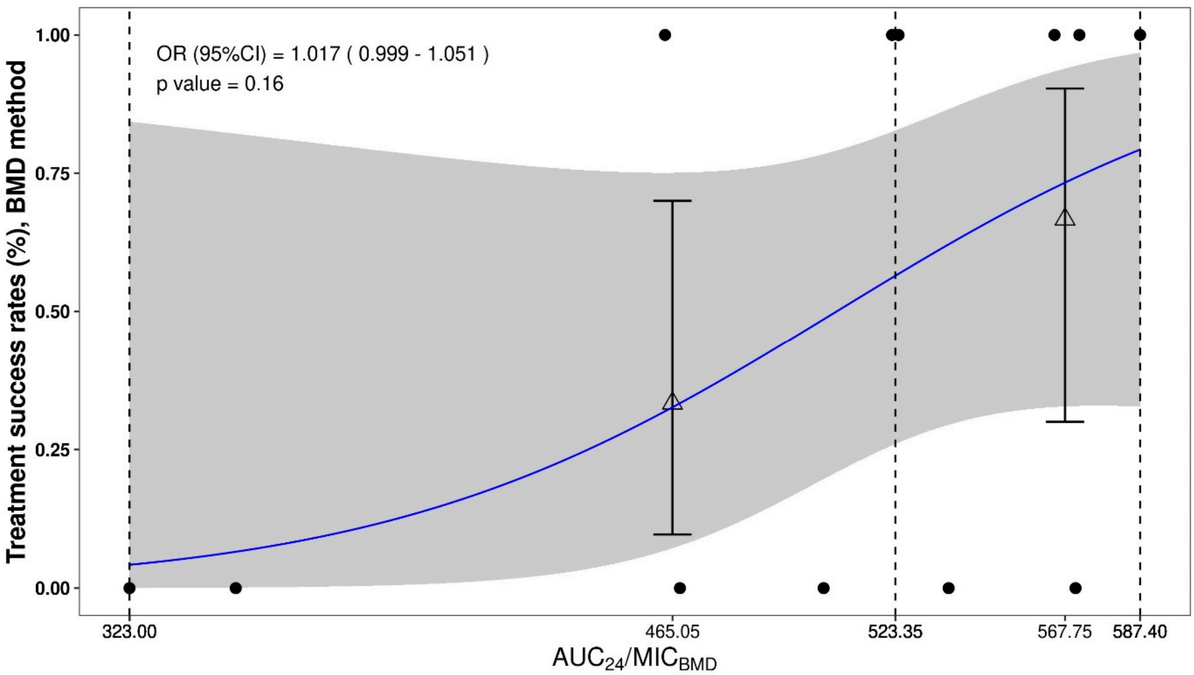

The upper and lower circles represent the presence or absence of treatment success across the range of vancomycin AUC_{24}/MIC_{BMD}, respectively. The dots depict the observed incidence for the quartiles of exposure, whereas the corresponding vertical bars represent the exact 95% CI calculated using Wilson's method. Finally, the middle line and its corresponding shaded area represent model-based exposure-efficacy relationship and the 95% CI respectively. Vertical dashed lines represent min, median and max percentile of AUC_{24}/MIC_{BMD}, respectively.

Figure S6. Correlation between Efficacy Outcomes and Trough Levels

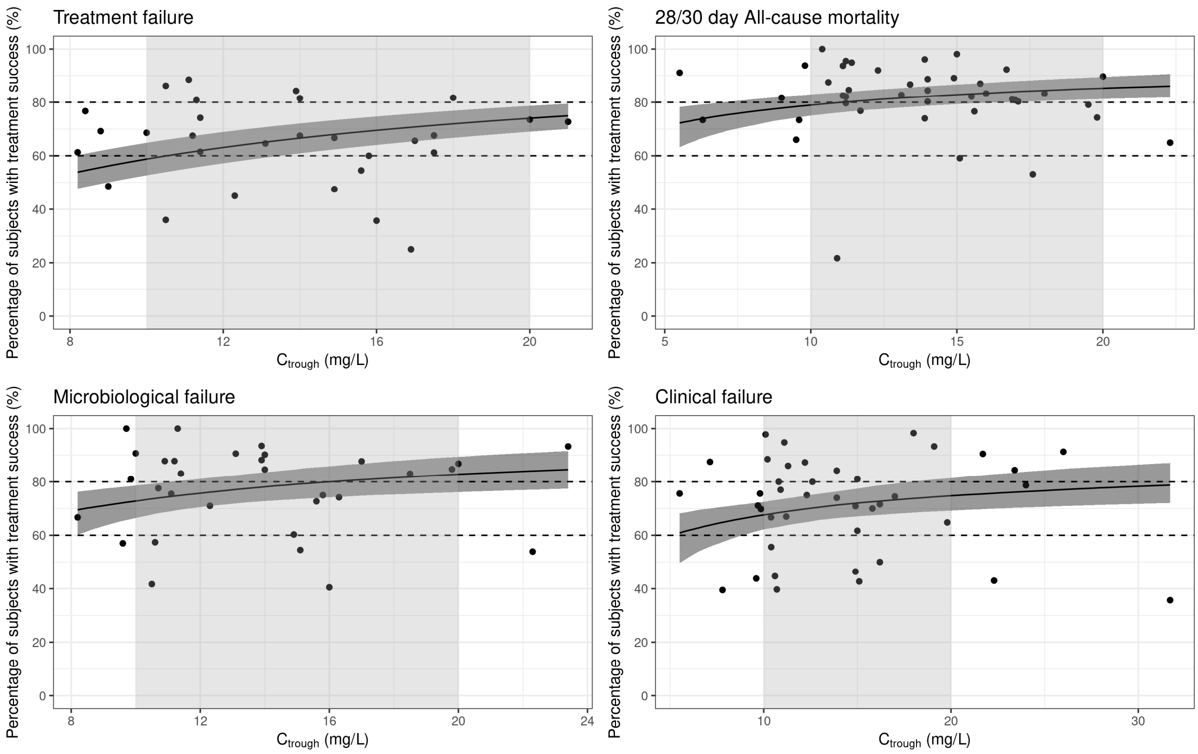

Black circles represent the observations from each study. Solid line and the shaded area represent the estimated Emax model curve with 95% credible intervals of parameters. The light grey shade represents the C_{trough} interval between 10 mg/L and 20 mg/L.

3: ARTICLES INCLUDED IN THE ANALYSIS

[1] Sohn Y, Rim JH, Cho Y, Hyun J, Baek Y, Kim M, et.al. Association of vancomycin trough concentration on the treatment outcome of patients with bacteremia caused by Enterococcus species. BMC Infect Dis. 2021 Oct 26;21(1):1099. doi: 10.1186/s12879-021-06809-x. PMID: 34702193; PMCID: PMC8547083.

[2] Marko R, Hajjar J, Nzeribe V, Pittman M, Deslandes V, Sant N, et.al. Therapeutic Drug Monitoring of Vancomycin in Adult Patients with Methicillin-Resistant Staphylococcus aureus Bacteremia or Pneumonia. Can J Hosp Pharm. 2021 Fall;74(4):334-343. doi: 10.4212/cjhp.v74i4.3195. PMID: 34602621; PMCID: PMC8463016.

[3] Katip W, Oberdorfer P. A Monocentric Retrospective Study of AUC/MIC Ratio of Vancomycin Associated with Clinical Outcomes and Nephrotoxicity in Patients with Enterococcal Infections. Pharmaceutics. 2021 Aug 31;13(9):1378. doi: 10.3390/pharmaceutics13091378. PMID: 34575453; PMCID: PMC8464995.

[4] Al-Sulaiti FK, Nader AM, Saad MO, Shaukat A, Parakadavathu R, Elzubair A, et.al. Clinical and Pharmacokinetic Outcomes of Peak-Trough-Based Versus Trough-Based Vancomycin Therapeutic Drug Monitoring Approaches: A Pragmatic Randomized Controlled Trial. Eur J Drug Metab Pharmacokinet. 2019 Oct;44(5):639-652. doi: 10.1007/s13318-019-00551-1. PMID: 30919233; PMCID: PMC6746691.

[5] Wan M, Walker SAN, Martin E, Elligsen M, Palmay L, Leis JA. The impact of vancomycin trough concentrations on outcomes in non-deep seated infections: a retrospective cohort study. BMC Pharmacol Toxicol. 2018 Jul 31;19(1):47. doi: 10.1186/s40360-018-0236-z. PMID: 30064515; PMCID: PMC6069851.

[6] Mogle BT, Steele JM, Seabury RW, Dang UJ, Kufel WD. Implementation of a two-point pharmacokinetic AUC-based vancomycin therapeutic drug monitoring approach in patients with methicillin-resistant Staphylococcus aureus bacteraemia. Int J Antimicrob Agents. 2018 Dec;52(6):805-810. doi: 10.1016/j.ijantimicag.2018.08.024. Epub 2018 Aug 31. PMID: 30176357.

[7] Jumah MTB, Vasoo S, Menon SR, De PP, Neely M, Teng CB. Pharmacokinetic/Pharmacodynamic Determinants of Vancomycin Efficacy in Enterococcal Bacteremia. Antimicrob Agents Chemother. 2018 Feb 23;62(3):e01602-17. doi: 10.1128/AAC.01602-17. PMID: 29263057; PMCID: PMC5826144.

[8] Fukumori S, Tsuji Y, Mizoguchi A, Kasai H, Ishibashi T, Iwamura N, et.al. Association of the clinical efficacy of vancomycin with the novel pharmacokinetic parameter area under the trough level (AUTL) in elderly patients with hospital-acquired pneumonia. J Clin Pharm Ther. 2016 Aug;41(4):399-402. doi: 10.1111/jcpt.12399. Epub 2016 May 4. PMID: 27144370.

[9] Suzuki Y, Tokimatsu I, Morinaga Y, Sato Y, Takano K, Kohno K, et.al. A retrospective analysis to estimate target trough concentration of vancomycin for febrile neutropenia in patients with hematological malignancy. Clin Chim Acta. 2015 Feb 2;440:183-7. doi: 10.1016/j.cca.2014.11.027. Epub 2014 Dec 2. PMID: 25476135.

[10] Zhang X, Wang D. The characteristics and impact indicator of vancomycin pharmacokinetics in cancer patients complicated with severe pneumonia. J Infect Chemother. 2020 May;26(5):492-497. doi: 10.1016/j.jiac.2019.12.019. Epub 2020 Jan 23. PMID: 31983615.

[11] Perin N, Roger C, Marin G, Molinari N, Evrard A, Lavigne JP, et.al. Vancomycin Serum Concentration after 48 h of Administration: A 3-Years Survey in an Intensive Care Unit. Antibiotics (Basel). 2020 Nov 10;9(11):793. doi: 10.3390/antibiotics9110793. PMID: 33182613; PMCID: PMC7698174.

[12] Yahav D, Abbas M, Nassar L, Ghrayeb A, Shepshelovich D, Kurnik D, et.al. Attention to age: similar dosing regimens lead to different vancomycin levels among older and younger patients. Age Ageing. 2019 Dec 1;49(1):26-31. doi: 10.1093/ageing/afz135. PMID: 31711101.

[13] Frazee E, Rule AD, Lieske JC, Kashani KB, Barreto JN, Virk A, et.al. Cystatin C-Guided Vancomycin Dosing in Critically Ill Patients: A Quality Improvement Project. Am J Kidney Dis. 2017 May;69(5):658-666. doi: 10.1053/j.ajkd.2016.11.016. Epub 2017 Jan 25. PMID: 28131530.

[14] Suzuki Y, Kawasaki K, Sato Y, Tokimatsu I, Itoh H, Hiramatsu K, et.al. Is peak concentration needed in therapeutic drug monitoring of vancomycin? A pharmacokinetic-pharmacodynamic analysis in patients with methicillin-resistant staphylococcus aureus pneumonia. Chemotherapy. 2012;58(4):308-12. doi: 10.1159/000343162. Epub 2012 Nov 7. PMID: 23147106.

[15] Wysocki M, Delatour F, Faurisson F, Rauss A, Pean Y, Misset B, et.al. Continuous versus intermittent infusion of vancomycin in severe Staphylococcal infections: prospective multicenter randomized study. Antimicrob Agents Chemother. 2001 Sep;45(9):2460-7. doi: 10.1128/AAC.45.9.2460-2467.2001. PMID: 11502515; PMCID: PMC90678.

[16] Gawronski KM, Goff DA, Brown J, Khadem TM, Bauer KA. A stewardship program's retrospective evaluation of vancomycin AUC24/MIC and time to microbiological clearance in patients with methicillin-resistant Staphylococcus aureus bacteremia and osteomyelitis. Clin Ther. 2013 Jun;35(6):772-9. doi: 10.1016/j.clinthera.2013.05.008. PMID: 23795575.

[17] Duszynska W, Taccone FS, Hurkacz M, Wiela-Hojenska A, Kübler A. Continuous vs. intermittent vancomycin therapy for Gram-positive infections not caused by methicillin-resistant Staphylococcus aureus. Minerva Anestesiol. 2016 Mar;82(3):284-93. Epub 2015 Jul 16. PMID: 26184702.

[18] Mizokami F, Shibasaki M, Yoshizue Y, Noro T, Mizuno T, Furuta K. Pharmacodynamics of vancomycin in elderly patients aged 75 years or older with methicillin-resistant Staphylococcus aureus hospital-acquired pneumonia. Clin Interv Aging. 2013;8:1015-21. doi: 10.2147/CIA.S50238. Epub 2013 Aug 7. PMID: 23966773; PMCID: PMC3743526.

[19] Zasowski EJ, Murray KP, Trinh TD, Finch NA, Pogue JM, Mynatt RP, et.al. Identification of Vancomycin Exposure-Toxicity Thresholds in Hospitalized Patients Receiving Intravenous Vancomycin. Antimicrob Agents Chemother. 2017 Dec 21;62(1):e01684-17. doi: 10.1128/AAC.01684-17. PMID: 29084753; PMCID: PMC5740375.

[20] Finch NA, Zasowski EJ, Murray KP, Mynatt RP, Zhao JJ, Yost R, et.al. A Quasi-Experiment To Study the Impact of Vancomycin Area under the Concentration-Time Curve-Guided Dosing on Vancomycin-Associated Nephrotoxicity. Antimicrob Agents Chemother. 2017 Nov 22;61(12):e01293-17. doi: 10.1128/AAC.01293-17. PMID: 28923869; PMCID: PMC5700348.

[21] Wang JL, Xue M, Wang HF, Huang LL, Li Q, Xu JY, et.al. [An area under curve-based nomogram to predicts vancomycin-associated nephrotoxicity in critically ill patients: a retrospective cohort study]. Zhonghua Nei Ke Za Zhi. 2022 Mar 1;61(3):291-297. Chinese. doi: 10.3760/cma.j.cn112138-20211011-00688. PMID: 35263970.

[22] Ueda T, Takesue Y, Nakajima K, Ichiki K, Ishikawa K, Yamada K, et.al. Validation of Vancomycin Area under the Concentration-Time Curve Estimation by the Bayesian Approach Using One-Point Samples for Predicting Clinical Outcomes in Patients with Methicillin-Resistant Staphylococcus aureus Infections. Antibiotics (Basel). 2022 Jan 13;11(1):96. doi: 10.3390/antibiotics11010096. PMID: 35052972; PMCID: PMC8772855.

[23] Yasu T, Konuma T, Oiwa-Monna M, Kato S, Isobe M, Takahashi S, et.al. Lower vancomycin trough levels in adults undergoing unrelated cord blood transplantation. Leuk Lymphoma. 2021 Feb;62(2):348-357. doi: 10.1080/10428194.2020.1834096. Epub 2020 Oct 25. PMID: 33100069.

[24] Wang Y, Dai N, Wei W, Jiang C. Outcomes and Nephrotoxicity Associated with Vancomycin Treatment in Patients 80 Years and Older. Clin Interv Aging. 2021 Jun 1;16:1023-1035. doi: 10.2147/CIA.S308878. PMID: 34103905; PMCID: PMC8179733.

[25] Liu K, Zhang Y, Xu X, Wu B, Ni J, Li T, et.al. Comparative Prevalence of Acute Kidney Injury in Chinese Patients Receiving Vancomycin with Concurrent β-Lactam Antibiotics: A Retrospective Cohort Study. Clin Ther. 2021 Oct;43(10):e319-e351. doi: 10.1016/j.clinthera.2021.08.008. Epub 2021 Sep 25. PMID: 34579971.

[26] Johnston MM, Huang V, Hall ST, Buckley MS, Bikin D, Barletta JF. Optimizing outcomes using vancomycin therapeutic drug monitoring in patients with MRSA bacteremia: trough concentrations or area under the curve? Diagn Microbiol Infect Dis. 2021 Oct;101(2):115442. doi: 10.1016/j.diagmicrobio.2021.115442. Epub 2021 May 28. PMID: 34192639.

[27] Al Sulaiman K, Alshaya A, Aljuhani O, Alsaeed A, Alshehri N, Vishwakarma R, et.al. The impact of early target attainment of vancomycin in critically ill patients with confirmed Gram-positive infection: A retrospective cohort study. BMC Infect Dis. 2021 Nov 24;21(1):1182. doi: 10.1186/s12879-021-06840-y. PMID: 34819023; PMCID: PMC8613993.

[28] Zhang Y, Wang T, Zhang D, You H, Dong Y, Liu Y, et.al. Therapeutic Drug Monitoring Coupled With Bayesian Forecasting Could Prevent Vancomycin-Associated Nephrotoxicity in Renal Insufficiency Patients: A Prospective Study and Pharmacoeconomic Analysis. Ther Drug Monit. 2020 Aug;42(4):600-609. doi: 10.1097/FTD.0000000000000750. PMID: 32097248.

[29] Ueki T, Sanematsu E, Furuya Y, Shinohara Y, Murakami Y, Miyazaki A, et.al. Relationship between vancomycin-associated nephrotoxicity and the number of combined nephrotoxic agents. Pharmazie. 2020 Jun 1;75(6):279-283. doi: 10.1691/ph.2020.0393. PMID: 32539926.

[30] Qin X, Tsoi MF, Zhao X, Zhang L, Qi Z, Cheung BMY. Vancomycin-associated acute kidney injury in Hong Kong in 2012-2016. BMC Nephrol. 2020 Feb 3;21(1):41. doi: 10.1186/s12882-020-1704-4. PMID: 32013870; PMCID: PMC6998253.

[31] Pan C, Wen A, Li X, Li D, Zhang Y, Liao Y, et.al. Development and Validation of a Risk Prediction Model of Vancomycin-Associated Nephrotoxicity in Elderly Patients: A Pilot Study. Clin Transl Sci. 2020 May;13(3):491-497. doi: 10.1111/cts.12731. Epub 2020 Jan 9. PMID: 31785129; PMCID: PMC7214653.

[32] Mcgrady KA, Benton M, Tart S, Bowers R. Evaluation of traditional initial vancomycin dosing versus utilizing an electronic AUC/MIC dosing program. Pharm Pract (Granada). 2020 Jul-Sep;18(3):2024. doi: 10.18549/PharmPract.2020.3.2024. Epub 2020 Sep 15. PMID: 33005260; PMCID: PMC7508472.

[33] Ma NH, Walker SAN, Elligsen M, Kiss A, Palmay L, Ho G, et.al. Retrospective multicentre matched cohort study comparing safety and efficacy outcomes of intermittent-infusion versus continuous-infusion vancomycin. J Antimicrob Chemother. 2020 Apr 1;75(4):1038-1046. doi: 10.1093/jac/dkz531. PMID: 31919504.

[34] Imai S, Takekuma Y, Kashiwagi H, Miyai T, Kobayashi M, Iseki K, et.al. Validation of the usefulness of artificial neural networks for risk prediction of adverse drug reactions used for individual patients in clinical practice. PLoS One. 2020 Jul 29;15(7):e0236789. doi: 10.1371/journal.pone.0236789. PMID: 32726360; PMCID: PMC7390378.

[35] Brunetti L, Song JH, Suh D, Kim HJ, Seong YH, Lee DS, et.al. The risk of vancomycin toxicity in patients with liver impairment. Ann Clin Microbiol Antimicrob. 2020 Mar 31;19(1):13. doi: 10.1186/s12941-020-00354-2. PMID: 32234065; PMCID: PMC7110653.

[36] Truong J, Smith SR, Veillette JJ, Forland SC. Individualized Pharmacokinetic Dosing of Vancomycin Reduces Time to Therapeutic Trough Concentrations in Critically Ill Patients. J Clin Pharmacol. 2018 Sep;58(9):1123-1130. doi: 10.1002/jcph.1273. Epub 2018 Jun 29. PMID: 29957824.

[37] Yahav D, Abbas M, Nassar L, Ghrayeb A, Kurnik D, Shepshelovich D, et.al. The association of vancomycin trough levels with outcomes among patients with methicillin-resistant Staphylococcus aureus (MRSA) infections: Retrospective cohort study. PLoS One. 2019 Apr 4;14(4):e0214309. doi: 10.1371/journal.pone.0214309. PMID: 30946754; PMCID: PMC6448937.

[38] de Almeida CDC, Simões E Silva AC, de Queiroz Oliveira JA, Batista ISF, Pereira FH, Gonçalves JE, et.al. Vancomycin-associated nephrotoxicity in non-critically ill patients admitted in a Brazilian public hospital: A prospective cohort study. PLoS One. 2019 Sep 5;14(9):e0222095. doi: 10.1371/journal.pone.0222095. PMID: 31487314; PMCID: PMC6728013.

[39] Nakashima T, Koido K, Baba H, Otsuka R, Okinaka K, Sano T, Nishigaki R, Hashimoto H, Otsuka T, Esaki M, Terakado H. Contribution of pharmacists with expertise in infectious diseases to appropriate individualized vancomycin dosing. Pharmazie. 2018 Jul 1;73(7):422-424. doi: 10.1691/ph.2018.8427. PMID: 30001779.

[40] May CC, Erwin BL, Childress M, Cortopassi J, Curtis G, Kilpatrick T, et.al. Assessment of acute kidney injury in neurologically and traumatically injured intensive care patients receiving large vancomycin doses. Int J Crit Illn Inj Sci. 2018 Oct-Dec;8(4):194-200. doi: 10.4103/IJCIIS.IJCIIS_39_18. PMID: 30662865; PMCID: PMC6311967.

[41] Liang X, Fan Y, Yang M, Zhang J, Wu J, Yu J, et.al. A Prospective Multicenter Clinical Observational Study on Vancomycin Efficiency and Safety With Therapeutic Drug Monitoring. Clin Infect Dis. 2018 Nov 13;67(suppl_2):S249-S255. doi: 10.1093/cid/ciy680. PMID: 30423040.

[42] Han Z, Pettit NN, Landon EM, Brielmaier BD. Impact of Pharmacy Practice Model Expansion on Pharmacokinetic Services: Optimization of Vancomycin Dosing and Improved Patient Safety. Hosp Pharm. 2017 Apr;52(4):273-279. doi: 10.1310/hpj5204-273. PMID: 28515506; PMCID: PMC5424831.

[43] Chavada R, Ghosh N, Sandaradura I, Maley M, Van Hal SJ. Establishment of an AUC0-24 Threshold for Nephrotoxicity Is a Step towards Individualized Vancomycin Dosing for Methicillin-Resistant Staphylococcus aureus Bacteremia. Antimicrob Agents Chemother. 2017 Apr 24;61(5):e02535-16. doi: 10.1128/AAC.02535-16. PMID: 28242672; PMCID: PMC5404579.

[44] Anderson CW, Cazares KS, Lustik MB, Patel SM, Denunzio TM. Vancomycin vs. Vancomycin/Piperacillin-Tazobactam-Associated Acute Kidney Injury in Noncritically Ill Patients at a Tertiary Care Military Treatment Facility. Mil Med. 2017 Sep;182(9):e1773-e1778. doi: 10.7205/MILMED-D-16-00197. PMID: 28885936.

[45] Hammoud K, Brimacombe M, Yu A, Goodloe N, Haidar W, El Atrouni W. Vancomycin Trough and Acute Kidney Injury: A Large Retrospective, Cohort Study. Am J Nephrol. 2016;44(6):456-461. doi: 10.1159/000452427. Epub 2016 Oct 28. PMID: 27788522.

[46] Hanrahan TP, Kotapati C, Roberts MJ, Rowland J, Lipman J, Roberts JA, et.al. Factors associated with vancomycin nephrotoxicity in the critically ill. Anaesth Intensive Care. 2015 Sep;43(5):594-9. doi: 10.1177/0310057X1504300507. PMID: 26310409.

[47] Dong MH, Wang JW, Wu Y, Chen BY, Yu M, Wen AD. Evaluation of body weight-based vancomycin therapy and the incidence of nephrotoxicity: a retrospective study in the northwest of China. Int J Infect Dis. 2015 Aug;37:125-8. doi: 10.1016/j.ijid.2015.06.025. Epub 2015 Jul 6. PMID: 26159843.

[48] Hanrahan TP, Harlow G, Hutchinson J, Dulhunty JM, Lipman J, Whitehouse T, et.al. Vancomycin-associated nephrotoxicity in the critically ill: a retrospective multivariate regression analysis*. Crit Care Med. 2014 Dec;42(12):2527-36. doi: 10.1097/CCM.0000000000000514. PMID: 25083977.

[49] Hall RG 2nd, Blaszczyk AT, Thompson KA, Brouse SD, Giuliano CA, Frei CR, et.al. Impact of empiric weight-based vancomycin dosing on nephrotoxicity and mortality in geriatric patients with methicillin-resistant Staphylococcus aureus bacteraemia. J Clin Pharm Ther. 2014 Dec;39(6):653-7. doi: 10.1111/jcpt.12203. Epub 2014 Sep 8. PMID: 25200273; PMCID: PMC4211949.

[50] Burgess LD, Drew RH. Comparison of the incidence of vancomycin-induced nephrotoxicity in hospitalized patients with and without concomitant piperacillin-tazobactam. Pharmacotherapy. 2014 Jul;34(7):670-6. doi: 10.1002/phar.1442. Epub 2014 May 22. PMID: 24855041.

[51] Mizuno T, Mizokami F, Fukami K, Ito K, Shibasaki M, Nagamatsu T, et.al. The influence of severe hypoalbuminemia on the half-life of vancomycin in elderly patients with methicillin-resistant Staphylococcus aureus hospital-acquired pneumonia. Clin Interv Aging. 2013;8:1323-8. doi: 10.2147/CIA.S52259. Epub 2013 Sep 30. PMID: 24109180; PMCID: PMC3793010.

[52] Horey A, Mergenhagen KA, Mattappallil A. The Relationship of nephrotoxicity to vancomycin trough serum concentrations in a veteran's population: a retrospective analysis. Ann Pharmacother. 2012 Nov;46(11):1477-83. doi: 10.1345/aph.1R158. Epub 2012 Oct 16. PMID: 23073306.

[53] Lodise TP, Patel N, Lomaestro BM, Rodvold KA, Drusano GL. Relationship between initial vancomycin concentration-time profile and nephrotoxicity among hospitalized patients. Clin Infect Dis. 2009 Aug 15;49(4):507-14. doi: 10.1086/600884. PMID: 19586413.

[54] Hidayat LK, Hsu DI, Quist R, Shriner KA, Wong-Beringer A. High-dose vancomycin therapy for methicillin-resistant Staphylococcus aureus infections: efficacy and toxicity. Arch Intern Med. 2006 Oct 23;166(19):2138-44. doi: 10.1001/archinte.166.19.2138. PMID: 17060545.

[55] Flannery AH, Delozier NL, Effoe SA, Wallace KL, Cook AM, Burgess DS. First-Dose Vancomycin Pharmacokinetics Versus Empiric Dosing on Area-Under-the-Curve Target Attainment in Critically Ill Patients. Pharmacotherapy. 2020 Dec;40(12):1210-1218. doi: 10.1002/phar.2486. Epub 2020 Dec 11. PMID: 33176005.

[56] Hirai T, Hanada K, Kanno A, Akashi M, Itoh T. Risk factors for vancomycin nephrotoxicity and time course of renal function during vancomycin treatment. Eur J Clin Pharmacol. 2019 Jun;75(6):859-866. doi: 10.1007/s00228-019-02648-7. Epub 2019 Feb 15. Erratum in: Eur J Clin Pharmacol. 2019 Mar 19;: PMID: 30770940.

[57] Okada N, Chuma M, Azuma M, Nakamura S, Miki H, Hamano H, et.al. Effect of serum concentration and concomitant drugs on vancomycin-induced acute kidney injury in haematologic patients: a single-centre retrospective study. Eur J Clin Pharmacol. 2019 Dec;75(12):1695-1704. doi: 10.1007/s00228-019-02756-4. Epub 2019 Sep 11. PMID: 31511938.

[58] Ramírez E, Jiménez C, Borobia AM, Tong HY, Medrano N, Krauel-Bidwell L, et.al. Vancomycin-induced acute kidney injury detected by a prospective pharmacovigilance program from laboratory signals. Ther Drug Monit. 2013 Jun;35(3):360-6. doi: 10.1097/FTD.0b013e318286eb86. PMID: 23666575.

[59] Molina KC, Barletta JF, Hall ST, Yazdani C, Huang V. The Risk of Acute Kidney Injury in Critically Ill Patients Receiving Concomitant Vancomycin With Piperacillin-Tazobactam or Cefepime. J Intensive Care Med. 2020 Dec;35(12):1434-1438. doi: 10.1177/0885066619828290. Epub 2019 Feb 10. PMID: 30741072.

[60] Sharma M, Braekevelt K, Kale-Pradhan P, Szpunar S, Khatib R. Are Blacks at Higher Risk for Vancomycin-Related Acute Kidney Injury? J Pharm Pract. 2020 Oct;33(5):592-597. doi: 10.1177/0897190018800093. Epub 2019 Jan 22. PMID: 30669919.

[61] Hays WB, Tillman E. Vancomycin-Associated Acute Kidney Injury in Critically Ill Adolescent and Young Adult Patients. J Pharm Pract. 2020 Dec;33(6):749-753. doi: 10.1177/0897190019829652. Epub 2019 Feb 26. PMID: 30808269.

[62] Covvey JR, Erickson O, Fiumara D, Mazzei K, Moszczenski Z, Slipak K, et.al. Comparison of Vancomycin Area-Under-the-Curve Dosing Versus Trough Target-Based Dosing in Obese and Nonobese Patients With Methicillin-Resistant Staphylococcus aureus Bacteremia. Ann Pharmacother. 2020 Jul;54(7):644-651. doi: 10.1177/1060028019897100. Epub 2019 Dec 30. PMID: 31888350.

[63] Zimmermann AE, Katona BG, Plaisance KI. Association of vancomycin serum concentrations with outcomes in patients with gram-positive bacteremia. Pharmacotherapy. 1995 Jan-Feb;15(1):85-91. PMID: 7739950.

[64] Brumer E, Dubrovskaya Y, Scipione MR, Aberle C, Rahimian J, Papadopoulos J. Evaluation of Treatment Courses When Vancomycin Is Given Every 8 Hours in Adult Patients. J Pharm Pract. 2015 Dec;28(6):511-7. doi: 10.1177/0897190014544817. Epub 2014 Aug 11. PMID: 25112304.

[65] Brown ML, Hutchison AM, McAtee AM, Gaillard PR, Childress DT. Allometric versus consensus guideline dosing in achieving target vancomycin trough concentrations. Am J Health Syst Pharm. 2017 Jul 15;74(14):1067-1075. doi: 10.2146/ajhp160260. Epub 2017 May 18. PMID: 28522643.

[66] Cano EL, Haque NZ, Welch VL, Cely CM, Peyrani P, Scerpella EG, et.al. Improving Medicine through Pathway Assessment of Critical Therapy of Hospital-Acquired Pneumonia (IMPACT-HAP) Study Group. Incidence of nephrotoxicity and association with vancomycin use in intensive care unit patients with pneumonia: retrospective analysis of the IMPACT-HAP Database. Clin Ther. 2012 Jan;34(1):149-57. doi: 10.1016/j.clinthera.2011.12.013. PMID: 22284995.

[67] Cappelletty D, Jablonski A, Jung R. Risk factors for acute kidney injury in adult patients receiving vancomycin. Clin Drug Investig. 2014 Mar;34(3):189-93. doi: 10.1007/s40261-013-0163-0. PMID: 24385282.

[68] Bhasin B, Ber Ce P, Szabo A, Chhabra S, D'Souza A. Correlates and Outcomes of Early Acute Kidney Injury after Hematopoietic Cell Transplantation. Am J Med Sci. 2021 Jul;362(1):72-77. doi: 10.1016/j.amjms.2021.03.013. Epub 2021 Apr 2. PMID: 33812909.

[69] Huang M, Wu H, Zhou J, Xu M, Zhou S. Efficacy of Vancomycin on Gram-Positive Bacterial Infection in Elderly Critical Patients and Risk Factors Associated With Nephrotoxicity. Arch Iran Med. 2018 Aug 1;21(8):349-355. PMID: 30113856.

[70] Higashi T, Tsukamoto H, Kodawara T, Igarashi T, Watanabe K, Yano R, et.al. Evaluation of risk factors for nephrotoxicity associated with high-dose vancomycin in Japanese patients. Pharmazie. 2021 Feb 25;76(2):114-118. doi: 10.1691/ph.2021.0138. PMID: 33714289.

[71] Fodero KE, Horey AL, Krajewski MP, Ruh CA, Sellick JA Jr, Mergenhagen KA. Impact of an Antimicrobial Stewardship Program on Patient Safety in Veterans Prescribed Vancomycin. Clin Ther. 2016 Mar;38(3):494-502. doi: 10.1016/j.clinthera.2016.01.001. Epub 2016 Jan 29. PMID: 26831569.

[72] Golenia BS, Levine AR, Moawad IM, Yeh DD, Arpino PA. Evaluation of a vancomycin dosing nomogram based on the Modification of Diet in Renal Disease equation in intensive care unit patients. J Crit Care. 2013 Oct;28(5):710-6. doi: 10.1016/j.jcrc.2013.01.004. Epub 2013 Mar 15. PMID: 23499418.

[73] Hale CM, Seabury RW, Steele JM, Darko W, Miller CD. Are Vancomycin Trough Concentrations of 15 to 20 mg/L Associated With Increased Attainment of an AUC/MIC ⩾ 400 in Patients With Presumed MRSA Infection? J Pharm Pract. 2017 Jun;30(3):329-335. doi: 10.1177/0897190016642692. Epub 2016 Apr 12. PMID: 27074786.

[74] Hall SF, Athans V, Wanek MR, Wang L, Estep JD, Williams B. Evaluation of a hospital-wide vancomycin-dosing nomogram in patients with continuous-flow left ventricular assist devices. Int J Artif Organs. 2021 Jun;44(6):411-417. doi: 10.1177/0391398820975037. Epub 2020 Nov 21. PMID: 33222593.

[75] Moh'd H, Kheir F, Kong L, Du P, Farag H, Mohamad A, et.al. Incidence and predictors of vancomycin-associated nephrotoxicity. South Med J. 2014 Jun;107(6):383-8. doi: 10.14423/01.SMJ.0000450716.84291.59. PMID: 24945176.

[76] Han HK, An H, Shin KH, Shin D, Lee SH, Kim JH, et.al. Trough concentration over 12.1 mg/L is a major risk factor of vancomycin-related nephrotoxicity in patients with therapeutic drug monitoring. Ther Drug Monit. 2014 Oct;36(5):606-11. doi: 10.1097/FTD.0000000000000061. PMID: 24577126.

[77] Haruki Y, Hagiya H, Haruki M, Inoue Y, Sugiyama T. Concomitant vancomycin and piperacillin/tazobactam treatment is associated with an increased risk of acute kidney injury in Japanese

patients. J Infect Chemother. 2020 Oct;26(10):1026-1032. doi: 10.1016/j.jiac.2020.05.012. Epub 2020 Jun 16. PMID: 32561128.

[78] Hermsen ED, Hanson M, Sankaranarayanan J, Stoner JA, Florescu MC, Rupp ME. Clinical outcomes and nephrotoxicity associated with vancomycin trough concentrations during treatment of deep-seated infections. Expert Opin Drug Saf. 2010 Jan;9(1):9-14. doi: 10.1517/14740330903413514. PMID: 20021290.

[79] Hong LT, Goolsby TA, Sherman DS, Mueller SW, Reynolds P, Cava L, et.al. Continuous infusion vs intermittent vancomycin in neurosurgical intensive care unit patients. J Crit Care. 2015 Oct;30(5):1153.e1-6. doi: 10.1016/j.jcrc.2015.06.012. Epub 2015 Jun 23. PMID: 26239323.

[80] Ghehi MT, Rezaee S, Hayatshahi A, Hadjibabaie M, Gholami K, Javadi M, et.al. Vancomycin Pharmacokinetic Parameters in Patients Undergoing Hematopoietic Stem Cell Transplantation (HSCT). Int J Hematol Oncol Stem Cell Res. 2013;7(4):1-9. PMID: 24505536; PMCID: PMC3915428.

[81] Imai S, Yamada T, Kasashi K, Kobayashi M, Iseki K. Usefulness of a decision tree model for the analysis of adverse drug reactions: Evaluation of a risk prediction model of vancomycin-associated nephrotoxicity constructed using a data mining procedure. J Eval Clin Pract. 2017 Dec;23(6):1240-1246. doi: 10.1111/jep.12767. Epub 2017 May 23. PMID: 28544476.

[82] Imai S, Yamada T, Kasashi K, Niinuma Y, Kobayashi M, Iseki K. Construction of a risk prediction model of vancomycin-associated nephrotoxicity to be used at the time of initial therapeutic drug monitoring: A data mining analysis using a decision tree model. J Eval Clin Pract. 2019 Feb;25(1):163-170. doi: 10.1111/jep.13039. Epub 2018 Oct 2. PMID: 30280456.

[83] Ko A, Harada MY, Barmparas G, Jay J, Sun BJ, Chen E, et.al. Reducing acute kidney injury due to vancomycin in trauma patients. J Trauma Acute Care Surg. 2016 Aug;81(2):352-7. doi: 10.1097/TA.0000000000001105. PMID: 27192471.

[84] Kullar R, Leonard SN, Davis SL, Delgado G Jr, Pogue JM, Wahby KA, et.al. Validation of the effectiveness of a vancomycin nomogram in achieving target trough concentrations of 15-20 mg/L suggested by the vancomycin consensus guidelines. Pharmacotherapy. 2011 May;31(5):441-8. doi: 10.1592/phco.31.5.441. PMID: 21923425.

[85] Kullar R, Davis SL, Taylor TN, Kaye KS, Rybak MJ. Effects of targeting higher vancomycin trough levels on clinical outcomes and costs in a matched patient cohort. Pharmacotherapy. 2012 Mar;32(3):195-201. doi: 10.1002/j.1875-9114.2011.01017.x. Erratum in: Pharmacotherapy. 2012 Sep;32(9):869. PMID: 22392452.

[86] Ley EJ, Liou DZ, Singer MB, Mirocha J, Srour M, Bukur M, et.al. Supratherapeutic vancomycin levels after trauma predict acute kidney injury and mortality. J Surg Res. 2013 Sep;184(1):501-6. doi: 10.1016/j.jss.2013.04.047. Epub 2013 May 11. PMID: 23731689.

[87] Liu Y, Yin Y, Liu XZ, Yao HJ, Li LX, Chen JH, et.al. Retrospective Analysis of Vancomycin Nephrotoxicity in Elderly Chinese Patients. Pharmacology. 2015;95(5-6):279-84. doi: 10.1159/000381783. Epub 2015 May 20. PMID: 25997622.

[88] Masuda N, Maiguma T, Komoto A, Haruki Y, Sugiyama T, Kondo S, et.al. Impact of pharmacist intervention on preventing nephrotoxicity from vancomycin. Int J Clin Pharmacol Ther. 2015 Apr;53(4):284-91. doi: 10.5414/CP202274. PMID: 25740266.

[89] Meng L, Wong T, Huang S, Mui E, Nguyen V, Espinosa G, et.al. Conversion from Vancomycin Trough Concentration-Guided Dosing to Area Under the Curve-Guided Dosing Using Two Sample Measurements in Adults: Implementation at an Academic Medical Center. Pharmacotherapy. 2019 Apr;39(4):433-442. doi: 10.1002/phar.2234. Epub 2019 Mar 18. PMID: 30739349.

[90] Muklewicz JD, Steuber TD, Edwards JD. Evaluation of area under the concentration-time curve-guided vancomycin dosing with or without piperacillin-tazobactam on the incidence of acute kidney injury. Int J Antimicrob Agents. 2021 Jan;57(1):106234. doi: 10.1016/j.ijantimicag.2020.106234. Epub 2020 Nov 21. PMID: 33232734.

[91] Oda K, Jono H, Nosaka K, Saito H. Reduced nephrotoxicity with vancomycin therapeutic drug monitoring guided by area under the concentration-time curve against a trough 15-20 μg/mL concentration. Int J Antimicrob Agents. 2020 Oct;56(4):106109. doi: 10.1016/j.ijantimicag.2020.106109. Epub 2020 Jul 25. PMID: 32721597.

[92] Park SJ, Lim NR, Park HJ, Yang JW, Kim MJ, Kim K, et.al. Evaluation of risk factors for vancomycin-induced nephrotoxicity. Int J Clin Pharm. 2018 Oct;40(5):1328-1334. doi: 10.1007/s11096-018-0634-8. Epub 2018 May 9. PMID: 29744794.

[93] Prabaker KK, Tran TP, Pratummas T, Goetz MB, Graber CJ. Elevated vancomycin trough is not associated with nephrotoxicity among inpatient veterans. J Hosp Med. 2012 Feb;7(2):91-7. doi: 10.1002/jhm.946. Epub 2011 Nov 15. PMID: 22086511.

[94] Pritchard L, Baker C, Leggett J, Sehdev P, Brown A, Bayley KB. Increasing vancomycin serum trough concentrations and incidence of nephrotoxicity. Am J Med. 2010 Dec;123(12):1143-9. doi: 10.1016/j.amjmed.2010.07.025. PMID: 21183005.

[95] Qian X, Du G, Weng C, Zhou H, Zhou X. Evaluation of the variability and safety of serum trough concentrations of vancomycin in patients admitted to the intensive care unit. Int J Infect Dis. 2017 Jul;60:17-22. doi: 10.1016/j.ijid.2017.04.018. Epub 2017 Apr 27. PMID: 28457752.

[96] Reynolds DC, Waite LH, Alexander DP, DeRyke CA. Performance of a vancomycin dosage regimen developed for obese patients. Am J Health Syst Pharm. 2012 Jun 1;69(11):944-50. doi: 10.2146/ajhp110324. PMID: 22610026.

[97] Robertson AD, Li C, Hammond DA, Dickey TA. Incidence of Acute Kidney Injury Among Patients Receiving the Combination of Vancomycin with Piperacillin-Tazobactam or Meropenem. Pharmacotherapy. 2018 Dec;38(12):1184-1193. doi: 10.1002/phar.2179. Epub 2018 Oct 3. PMID: 30175410.

[98] Rybak MJ, Albrecht LM, Boike SC, Chandrasekar PH. Nephrotoxicity of vancomycin, alone and with an aminoglycoside. J Antimicrob Chemother. 1990 Apr;25(4):679-87. doi: 10.1093/jac/25.4.679. PMID: 2351627.

[99] Sazanami K, Inose R, Dote S, Horiuchi N, Kobayashi Y, Muraki Y. Combination therapy of vancomycin and piperacillin/tazobactam in adult febrile neutropenia patients with haematopoietic malignancies increases the risk of acute kidney injury regardless of vancomycin trough concentration. J Chemother. 2021 Oct;33(6):440-442. doi: 10.1080/1120009X.2020.1848298. Epub 2020 Nov 23. PMID: 33222657.

[100] Alosaimy S, Murray KP, Zasowski EJ, Morrisette T, Lagnf AM, Lodise TP, et.al. Vancomycin Area Under the Curve to Predict Timely Clinical Response in the Treatment of Methicillin-resistant Staphylococcus aureus Complicated Skin and Soft Tissue Infections. Clin Infect Dis. 2021 Dec 6;73(11):e4560-e4567. doi: 10.1093/cid/ciaa1039. PMID: 32716506; PMCID: PMC8662764.

[101] Fan YX, Chen MT, Li NY, Liu XF, Yang MJ, Chen YC, et.al. Sequence Type 5 (ST5) as a Possible Predictor of Bacterial Persistence in Adult Patients with Methicillin-Resistant Staphylococcus aureus Pneumonia Treated with Vancomycin. Microbiol Spectr. 2022 Oct 26;10(5):e0134822. doi: 10.1128/spectrum.01348-22. Epub 2022 Sep 12. PMID: 36094217; PMCID: PMC9603198.

[102] Ren J, Hou Y, Li J, Gao Y, Li R, Jin X, et.al. An evaluation on the association of vancomycin trough concentration with mortality in critically ill patients: A multicenter retrospective study. Clin Transl Sci.

2021 Sep;14(5):1780-1790. doi: 10.1111/cts.13020. Epub 2021 Apr 9. PMID: 33835715; PMCID: PMC8504840.

[103] Lines J, Burchette J, Kullab SM, Lewis P. Evaluation of a trough-only extrapolated area under the curve vancomycin dosing method on clinical outcomes. Int J Clin Pharm. 2021 Feb;43(1):263-269. doi: 10.1007/s11096-020-01157-3. Epub 2020 Sep 23. PMID: 32964405.

[104] Hou Y, Ren J, Li J, Jin X, Gao Y, Li R, et.al. Relationship Between Mean Vancomycin Trough Concentration and Mortality in Critically Ill Patients: A Multicenter Retrospective Study. Front Pharmacol. 2021 Jul 19;12:690157. doi: 10.3389/fphar.2021.690157. PMID: 34349650; PMCID: PMC8326564.

[105] Lodise TP, Rosenkranz SL, Finnemeyer M, Evans S, Sims M, Zervos MJ, et.al. The Emperor's New Clothes: PRospective Observational Evaluation of the Association Between Initial VancomycIn Exposure and Failure Rates Among ADult HospitalizEd Patients With Methicillin-resistant Staphylococcus aureus Bloodstream Infections (PROVIDE). Clin Infect Dis. 2020 Apr 10;70(8):1536-1545. doi: 10.1093/cid/ciz460. PMID: 31157370; PMCID: PMC7145993.

[106] Chattaweelarp T, Changpradub D, Punyawudho B, Thunyaharn S, Santimaleeworagun W. Is Early Monitoring Better? Impact of Early Vancomycin Exposure on Treatment Outcomes and Nephrotoxicity in Patients with Methicillin-Resistant Staphylococcus aureus Infections. Antibiotics (Basel). 2020 Oct 4;9(10):672. doi: 10.3390/antibiotics9100672. PMID: 33020463; PMCID: PMC7601693.

[107] Makmor-Bakry M, Ahmat A, Shamsuddin A, Lau CL, Ramli R. Association between single trough-based area under the curve estimation of vancomycin and treatment outcome among methicillin-resistant Staphylococcus aureus bacteremia patients. Anaesthesiol Intensive Ther. 2019;51(3):218-223. doi: 10.5114/ait.2019.87362. PMID: 31434472.

[108] Clark L, Skrupky LP, Servais R, Brummitt CF, Dilworth TJ. Examining the Relationship Between Vancomycin Area Under the Concentration Time Curve and Serum Trough Levels in Adults With Presumed or Documented Staphylococcal Infections. Ther Drug Monit. 2019 Aug;41(4):483-488. doi: 10.1097/FTD.0000000000000622. PMID: 30817704.

[109] Shen K, Yang M, Fan Y, Liang X, Chen Y, Wu J, et.al. Model-based Evaluation of the Clinical and Microbiological Efficacy of Vancomycin: A Prospective Study of Chinese Adult In-house Patients. Clin Infect Dis. 2018 Nov 13;67(suppl_2):S256-S262. doi: 10.1093/cid/ciy667. PMID: 30423042.

[110] Komoto A, Maiguma T, Teshima D, Sugiyama T, Haruki Y. Effects of pharmacist intervention in Vancomycin treatment for patients with bacteremia due to Methicillin-resistant Staphylococcus aureus. PLoS One. 2018 Sep 6;13(9):e0203453. doi: 10.1371/journal.pone.0203453. PMID: 30188918; PMCID: PMC6126860.

[111] Fu CF, Huang JD, Wang JT, Lin SW, Wu CC. The ratio of pre-dialysis vancomycin trough serum concentration to minimum inhibitory concentration is associated with treatment outcomes in methicillin-resistant Staphylococcus aureus bacteremia. PLoS One. 2018 Mar 5;13(3):e0193585. doi: 10.1371/journal.pone.0193585. PMID: 29505620; PMCID: PMC5837094.

[112] Moise PA, Culshaw DL, Wong-Beringer A, Bensman J, Lamp KC, Smith WJ, et.al. Comparative Effectiveness of Vancomycin Versus Daptomycin for MRSA Bacteremia With Vancomycin MIC >1 mg/L: A Multicenter Evaluation. Clin Ther. 2016 Jan 1;38(1):16-30. doi: 10.1016/j.clinthera.2015.09.017. Epub 2015 Nov 14. PMID: 26585355.

[113] Ji M, Kim HK, Kim SK, Lee W, Sung H, Chun S, et.al. Vancomycin AUC24 /MIC Ratio in Patients with Methicillin-Resistant Staphylococcus aureus Pneumonia. J Clin Lab Anal. 2016 Sep;30(5):485-9. doi: 10.1002/jcla.21883. Epub 2015 Oct 26. PMID: 26499918; PMCID: PMC6807131.

[114] Stevenson S, Tang W, Cho Y, Mudge DW, Hawley CM, Badve SV, et.al. The role of monitoring vancomycin levels in patients with peritoneal dialysis-associated peritonitis. Perit Dial Int. 2015 Mar-

Apr;35(2):222-8. doi: 10.3747/pdi.2013.00156. Epub 2014 Mar 1. PMID: 24584597; PMCID: PMC4406318.

[115] Song KH, Kim HB, Kim HS, Lee MJ, Jung Y, Kim G, et.al. Impact of area under the concentration-time curve to minimum inhibitory concentration ratio on vancomycin treatment outcomes in methicillin-resistant Staphylococcus aureus bacteraemia. Int J Antimicrob Agents. 2015 Dec;46(6):689-95. doi: 10.1016/j.ijantimicag.2015.09.010. Epub 2015 Oct 17. PMID: 26555059.

[116] Casapao AM, Lodise TP, Davis SL, Claeys KC, Kullar R, Levine DP, et.al. Association between vancomycin day 1 exposure profile and outcomes among patients with methicillin-resistant Staphylococcus aureus infective endocarditis. Antimicrob Agents Chemother. 2015;59(6):2978-85. doi: 10.1128/AAC.03970-14. Epub 2015 Mar 9. PMID: 25753631; PMCID: PMC4432113.

[117] Cao G, Liang X, Zhang J, Zhou Y, Wu J, Zhang Y, et.al. Vancomycin serum trough concentration vs. clinical outcome in patients with gram-positive infection: a retrospective analysis. J Clin Pharm Ther. 2015 Dec;40(6):640-4. doi: 10.1111/jcpt.12323. Epub 2015 Sep 18. PMID: 26383121.

[118] Lodise TP, Drusano GL, Zasowski E, Dihmess A, Lazariu V, Cosler L, et.al. Vancomycin exposure in patients with methicillin-resistant Staphylococcus aureus bloodstream infections: how much is enough? Clin Infect Dis. 2014 Sep 1;59(5):666-75. doi: 10.1093/cid/ciu398. Epub 2014 May 27. PMID: 24867791.

[119] Lin Z, Jiang Z, Chen J, Ouyang B, Chen M, Guan X. [Clinical research for trough value of serum vancomycin in critical patients]. Zhonghua Wei Zhong Bing Ji Jiu Yi Xue. 2014 Jul;26(7):473-7. Chinese. doi: 10.3760/cma.j.issn.2095-4352.2014.07.006. PMID: 25027425.

[120] Jung Y, Song KH, Cho Je, Kim HS, Kim NH, Kim TS, et.al. Area under the concentration-time curve to minimum inhibitory concentration ratio as a predictor of vancomycin treatment outcome in methicillin-resistant Staphylococcus aureus bacteraemia. Int J Antimicrob Agents. 2014 Feb;43(2):179-83. doi: 10.1016/j.ijantimicag.2013.10.017. Epub 2013 Nov 18. PMID: 24315788.

[121] Ghosh N, Chavada R, Maley M, van Hal SJ. Impact of source of infection and vancomycin AUC0-24/MICBMD targets on treatment failure in patients with methicillin-resistant Staphylococcus aureus bacteraemia. Clin Microbiol Infect. 2014 Dec;20(12):O1098-105. doi: 10.1111/1469-0691.12695. Epub 2014 Jul 12. PMID: 24890030.

[122] Zelenitsky S, Rubinstein E, Ariano R, Iacovides H, Dodek P, Mirzanejad Y, Kumar A; Cooperative Antimicrobial Therapy of Septic Shock-CATSS Database Research Group. Vancomycin pharmacodynamics and survival in patients with methicillin-resistant Staphylococcus aureus-associated septic shock. Int J Antimicrob Agents. 2013 Mar;41(3):255-60. doi: 10.1016/j.ijantimicag.2012.10.015. Epub 2013 Jan 9. PMID: 23312606.

[123] Holmes NE, Turnidge JD, Munckhof WJ, Robinson JO, Korman TM, O'Sullivan MV, et.al. Vancomycin AUC/MIC ratio and 30-day mortality in patients with Staphylococcus aureus bacteremia. Antimicrob Agents Chemother. 2013 Apr;57(4):1654-63. doi: 10.1128/AAC.01485-12. Epub 2013 Jan 18. PMID: 23335735; PMCID: PMC3623342.

[124] Moore CL, Osaki-Kiyan P, Haque NZ, Perri MB, Donabedian S, Zervos MJ. Daptomycin versus vancomycin for bloodstream infections due to methicillin-resistant Staphylococcus aureus with a high vancomycin minimum inhibitory concentration: a case-control study. Clin Infect Dis. 2012 Jan 1;54(1):51-8. doi: 10.1093/cid/cir764. Epub 2011 Nov 21. PMID: 22109947.

[125] Cheong JY, Makmor-Bakry M, Lau CL, Abdul Rahman R. The relationship between trough concentration of vancomycin and effect on methicillin-resistant Staphylococcus aureus in critically ill patients. S Afr Med J. 2012 May 25;102(7):616-9. doi: 10.7196/samj.5343. PMID: 22748440.

[126] Brown J, Brown K, Forrest A. Vancomycin AUC24/MIC ratio in patients with complicated bacteremia and infective endocarditis due to methicillin-resistant Staphylococcus aureus and its association

with attributable mortality during hospitalization. Antimicrob Agents Chemother. 2012 Feb;56(2):634-8. doi: 10.1128/AAC.05609-11. Epub 2011 Nov 28. PMID: 22123681; PMCID: PMC3264249.

[127] Clemens EC, Chan JD, Lynch JB, Dellit TH. Relationships between vancomycin minimum inhibitory concentration, dosing strategies, and outcomes in methicillin-resistant Staphylococcus aureus bacteremia. Diagn Microbiol Infect Dis. 2011 Dec;71(4):408-14. doi: 10.1016/j.diagmicrobio.2011.08.002. Epub 2011 Sep 15. PMID: 21924852.

[128] Chung J, Oh JM, Cho EM, Jang HJ, Hong SB, Lim CM, Koh YS. Optimal dose of vancomycin for treating methicillin-resistant Staphylococcus aureus pneumonia in critically ill patients. Anaesth Intensive Care. 2011 Nov;39(6):1030-7. doi: 10.1177/0310057X1103900608. PMID: 22165354.

[129] Chan JD, Pham TN, Wong J, Hessel M, Cuschieri J, Neff M, et.al. Clinical outcomes of linezolid vs vancomycin in methicillin-resistant Staphylococcus aureus ventilator-associated pneumonia: retrospective analysis. J Intensive Care Med. 2011 Nov-Dec;26(6):385-91. doi: 10.1177/0885066610392893. Epub 2011 May 23. PMID: 21606058.

[130] Kullar R, Davis SL, Levine DP, Rybak MJ. Impact of vancomycin exposure on outcomes in patients with methicillin-resistant Staphylococcus aureus bacteremia: support for consensus guidelines suggested targets. Clin Infect Dis. 2011 Apr 15;52(8):975-81. doi: 10.1093/cid/cir124. PMID: 21460309.

[131] Mohammedi I, Descloux E, Argaud L, Le Scanff J, Robert D. Loading dose of vancomycin in critically ill patients: 15 mg/kg is a better choice than 500 mg. Int J Antimicrob Agents. 2006 Mar;27(3):259-62. doi: 10.1016/j.ijantimicag.2005.11.009. Epub 2006 Feb 10. PMID: 16472993.

[132] Jeffres MN, Isakow W, Doherty JA, McKinnon PS, Ritchie DJ, Micek ST, et.al. Predictors of mortality for methicillin-resistant Staphylococcus aureus health-care-associated pneumonia: specific evaluation of vancomycin pharmacokinetic indices. Chest. 2006 Oct;130(4):947-55. doi: 10.1378/chest.130.4.947. PMID: 17035423.

[133] Moise-Broder PA, Forrest A, Birmingham MC, Schentag JJ. Pharmacodynamics of vancomycin and other antimicrobials in patients with Staphylococcus aureus lower respiratory tract infections. Clin Pharmacokinet. 2004;43(13):925-42. doi: 10.2165/00003088-200443130-00005. PMID: 15509186.

[134] Vuagnat A, Stern R, Lotthe A, Schuhmacher H, Duong M, Hoffmeyer P, et.al. High dose vancomycin for osteomyelitis: continuous vs. intermittent infusion. J Clin Pharm Ther. 2004 Aug;29(4):351-7. doi: 10.1111/j.1365-2710.2004.00572.x. PMID: 15271102.

[135] Moise PA, Forrest A, Bhavnani SM, Birmingham MC, Schentag JJ. Area under the inhibitory curve and a pneumonia scoring system for predicting outcomes of vancomycin therapy for respiratory infections by Staphylococcus aureus. Am J Health Syst Pharm. 2000 Oct 15;57 Suppl 2:S4-9. doi: 10.1093/ajhp/57.suppl_2.S4. Erratum in: Am J Health Syst Pharm 2001 Jan 1;58(1):78. PMID: 11057360.

[136] Karam CM, McKinnon PS, Neuhauser MM, Rybak MJ. Outcome assessment of minimizing vancomycin monitoring and dosing adjustments. Pharmacotherapy. 1999 Mar;19(3):257-66. doi: 10.1592/phco.19.4.257.30933. Erratum in: Pharmacotherapy 1999 May;19(5):674. PMID: 10221365.

[137] Rojas L, Bunsow E, Muñoz P, Cercenado E, Rodríguez-Créixems M, Bouza E. Vancomycin MICs do not predict the outcome of methicillin-resistant Staphylococcus aureus bloodstream infections in correctly treated patients. J Antimicrob Chemother. 2012 Jul;67(7):1760-8. doi: 10.1093/jac/dks128. Epub 2012 May 3. PMID: 22556382.
